# Advancing soundscape assessment in open-plan offices: Insights from expert focus groups

**DOI:** 10.1177/1351010X251340905

**Published:** 2025-06-13

**Authors:** Zulfi Rachman, Francesco Aletta, Jian Kang

**Affiliations:** UCL Institute for Environmental Design and Engineering, The Bartlett, University College London (UCL), London, UK

**Keywords:** Indoor soundscape, open-plan office, focus group discussion, thematic analysis, questionnaires

## Abstract

Open-plan offices are designed to foster collaboration but often face challenges related to noise and acoustic comfort, which can affect employee well-being and productivity. The soundscape concept offers a holistic approach to assessing and improving these environments, yet existing methods often fall short of capturing the complexity of open-plan offices. This study addresses this gap by developing a structured questionnaire tailored for open-plan offices, integrating acoustic and non-acoustic factors. Expert focus group discussions were conducted to identify and expand upon key factors influencing soundscape assessments, building on insights from prior research. Thematic analysis revealed critical considerations, including individual characteristics, spatial dynamics, sound environment perception, office acoustic metrics, noise control, work performance, and psychosocial conditions. To effectively incorporate these elements, the study recommends adapting existing soundscape frameworks to the specific context of open-plan offices. The proposed approach prioritises brevity, logical structure, and user-friendly language to enhance participant engagement and data quality, enabling practical and insightful assessments centred on user experience.

## Introduction

The concept of open-plan offices was first introduced in the 1950s and reached peak popularity in the early 1970s.^1^ It remains widely used today due to the assumption that it enhances environmental design, fosters communication and boosts employee productivity.^2,3^ Additionally, open-plan layouts offer economic benefits such as increased space utilisation, higher occupant density and greater flexibility for reconfiguration.^4,5^ However, open-plan offices often present notable acoustical challenges, including issues with privacy, noise levels, interruptions and distractions.^6–9^ These issues can lead to more serious consequences, as noise-related problems have been shown to increase fatigue, disturbance, stress, annoyance and reduce productivity.^10–15^

While initially applied in outdoor contexts, the soundscape concept has recently gained significant traction as a framework for assessing and improving acoustic conditions in indoor environments,^16,17^ including office environments.^18–25^ Unlike traditional acoustic metrics that focus on objective measures, soundscape assessments adopt a holistic approach, incorporating subjective evaluations of both acoustic and non-acoustic factors.^26,27^ These factors directly influence how employees perceive and interact with their acoustic surroundings, ultimately shaping the overall workspace quality.

Building on this conceptual foundation, many soundscape assessments in open-plan offices have adopted the ISO 12913 framework.^20,24,25,28,29^ Although adapted for open-plan office settings, its application reveals limitations constraining a comprehensive understanding of the office soundscapes. While helpful, the Pleasantness and Eventfulness dimensions often fall short of capturing the complex interplay between acoustic conditions and work-related activities.^25,30^

Another framework frequently used in soundscape assessments for open-plan offices is ISO 22955.^14,31,32^ However, this standard lacks detailed methodologies for perceptual evaluations of open-plan office acoustics. While it provides examples of user surveys for assessing open-plan office acoustics, it does not offer specific guidance on data analysis procedures,^25^ limiting its practicality for deriving actionable conclusions.

Therefore, a refinement of soundscape assessment methods for open-plan offices is essential. A holistic integration of acoustic and non-acoustic factors is required to reflect diverse elements influencing occupants’ acoustic experiences in open-plan office environments. This integration aims to develop a more comprehensive and relevant soundscape assessment method, accurately reflecting the range of activities occurring within the workplace.

**Figure 1.**
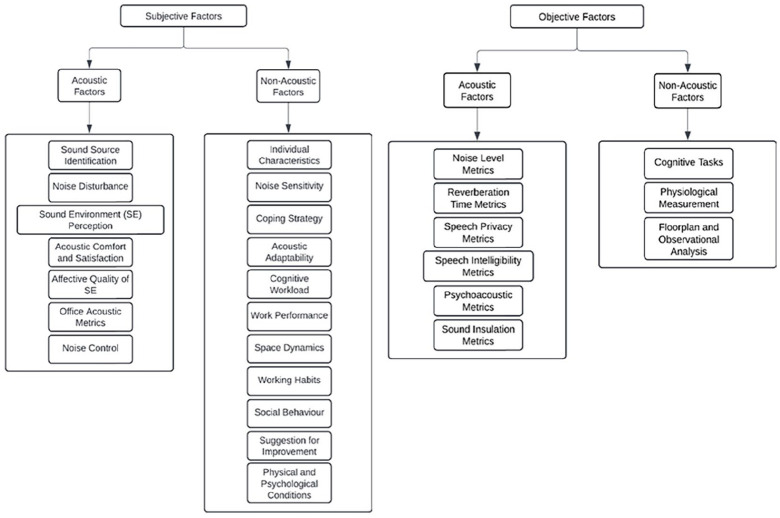
Subjective and objective factors in soundscape assessment in office environments.

To address these limitations and gaps, a systematic review of existing office soundscape assessment methods was previously conducted.^27^ This review analysed 41 studies to identify the methodologies and existing factors employed in soundscape assessments for office environments. The findings revealed that the most commonly used methods were subjective evaluations through questionnaires (*n* = 36) and objective evaluations via direct acoustic measurements (*n* = 28). These methodologies were collected and categorised into subjective and objective assessments. Each assessment item was extracted and analysed, resulting in the identification of recurring themes. These themes were subsequently grouped into acoustic and non-acoustic factors, forming a comprehensive framework for understanding the multidimensional nature of soundscape perception in office environments. The details of these factors are presented in Figure 1.

These findings highlighted the complex interdependencies between acoustic and non-acoustic influences, collectively impacting occupants’ experiences and performance in open-plan offices. Building on the systematic review findings, the next stage of this research involves a focus group discussion (FGD) with domain experts to gather diverse perspectives and collaboratively refine complex concepts. This approach is particularly valuable for critically evaluating and improving the existing framework in response to the research questions outlined below:

a. Are there any additional factors that should be considered to enhance the soundscape assessment framework for open-plan offices?
b. Which factors should be prioritised in soundscape assessments of open-plan offices? Are there any factors that are less relevant and should be excluded?
c. Are there specific factors to consider when designing an effective soundscape assessment tool for open-plan offices?
d. How can experts’ insights inform the development of effective soundscape assessment tools for open-plan offices?

By addressing these questions, this study aims to advance soundscape assessment methodologies for open-plan offices, providing a more comprehensive and practical framework, along with actionable tools like questionnaires for researchers, designers and policymakers.

## Materials and methods

### Expert focus groups

Focus groups are an effective tool for exploratory research, offering a structured yet adaptable approach to gathering in-depth data through participant interaction. This method enables researchers to uncover insights that may not emerge through other techniques, as the dynamic exchange of ideas encourages participants to share diverse perspectives, fostering a deeper understanding of the subject matter.^33,34^ In soundscape studies, focus groups are widely used to support concept refinement, standard development, translation, reflection and validation processes.^35–40^ This method aligns with the primary aim of the present study by exploring key factors for inclusion in soundscape assessments for open-plan offices. A previous systematic review identified a range of existing factors, categorised into acoustic and non-acoustic groups, which form the foundation for further exploration.^27^

### Participants

Participants for the focus group discussion (FGD) were selected using purposive sampling, a qualitative research strategy where individuals are intentionally chosen for their relevance to the study objectives.^41^ Experts in building acoustics, soundscape, workplace studies and building management personnel were invited to provide diverse and relevant perspectives during the discussions. We targeted a maximum of eight participants to ensure an effective and engaging discussion, as larger groups can be challenging to manage and hinder individual contributions.^33^

Over 20 experts were invited via email to mitigate potential no-shows,^34^ accompanied by a Participant Information Sheet (PIS) developed by following the Bartlett School of Environment, Energy and Resources (BSEER) ethics guidelines at University College London (UCL). The document outlined the session’s purpose, information about the researchers, the reason for their invitation, potential benefits and drawbacks of participation, data protection measures and the intended outcomes of the session.

This process led to a final group comprising lecturers, researchers and consultants from institutions in the UK, the Netherlands, Italy and Indonesia (see Table 1). Their diverse perspectives and extensive expertise enriched the discussions, facilitating a comprehensive exploration of the research themes.

**Table 1.** Overview of experts.

Expert	Country	Main areas of interest
1	Italy	Indoor soundscape, architectural engineering, indoor environmental quality and multi-domain studies
2	England	Office soundscapes, virtual evaluation of soundscapes and immersive audio applications
3	Italy	Office soundscapes, virtual reality environments, biophilic design, energy-efficient behaviour and indoor environmental quality
4	England	Indoor soundscapes, acoustics in historical spaces, open-plan office soundscapes and machine learning in acoustics
5	Indonesia	Soundscape, acoustics, psychoacoustics and audio engineering
6	Indonesia	Room acoustics, building physics, soundscape and psychoacoustics
7	England	Open-plan office soundscapes, acoustic design in schools and soundscape management
8	Netherlands	Workplace strategy, corporate real estate management and employee well-being

Upon confirming participation, attendees were invited to complete a scheduling poll via Doodle Premium, which ensured anonymity while identifying a mutually convenient session time. The session was scheduled for November 12, 2024, from 1:00 to 2:30 PM (GMT 0). Preparatory materials, which included a summary of the systematic review that informed the discussion topics, were distributed alongside the consent form.^27^ These materials provided participants with the necessary context for effective engagement. Participants were required to review, sign and return the consent form before the session. This study was approved as ‘low risk’ by the BSEER Local Research Ethics Committee at UCL, affirming its adherence to ethical research standards.

### Focus group setting and procedure

The Focus Group Discussion (FGD) was conducted online and moderated by the researcher (ZR). The session, lasting approximately 90 min, employed a structured question protocol to guide the discussion and ensure comprehensive coverage of the topics. Mentimeter, a web-based interactive platform, was used to collect and display participants’ responses in real-time. This tool enhanced the session’s interactive nature, allowing the moderator to investigate key insights as they emerged. The combination of a structured protocol and interactive tools ensured the discussion remained focussed while encouraging active participant engagement.

**Figure 2.**
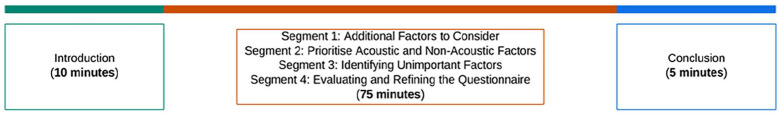
Session structure.

As shown in Figure 2, the session was divided into several segments, starting with a 10-min introduction to familiarise participants with the session’s objectives and tools. This was followed by a 75-min main discussion and a 5-min wrap-up summarising key takeaways. The following key questions guided the discussion:

a. What additional factors, beyond those listed in the Supplemental Materials, should be considered in an office soundscape assessment and why are these factors important?
b. Which factors most significantly influence the soundscape of open-plan offices and how should they be prioritised?
c. Which acoustic and non-acoustic factors are deemed irrelevant for office soundscape assessment and what justifies their exclusion?
d. How can the identified acoustic and non-acoustic factors be effectively incorporated into the design of a soundscape questionnaire?

### Data analysis

The discussion was recorded and transcribed verbatim to ensure authenticity and preserve the richness of participant responses. To analyse the data, this study employed thematic analysis, a widely used method in soundscape research that supports the organisation and interpretation of insights derived from participant opinions. This approach offers a flexible yet structured framework for identifying, analysing and reporting patterns across the dataset.^17,42–47^

The analysis process followed six key steps: (1) Familiarising with the data, (2) Generating initial codes, (3) Searching for themes, (4) Reviewing themes, (5) Defining and naming themes, and (6) Producing the final report.^48^ These steps were carried out manually using NVivo14 software to ensure precision and consistency. For instance, during the coding process, responses related to affective dimensions were assigned an initial code, which was then grouped with similar responses to form the theme Perceived Soundscape Quality. NVivo14 facilitated efficient data management and helped identify patterns, which were later visualised using a separate diagramming tool. Two coauthors independently reviewed the codes and themes to ensure reliability and collaboratively resolved discrepancies. Table 2 illustrates an example of this process in practice.

**Table 2.** Example of the coding process.

Example of excerpt	Code	Sub-theme	Theme
‘Pleasantness and eventfulness appear to be useful in open-plan office environments . . .’	Affective Dimensions	Perceived affective quality	Holistic Approach to Office Soundscape Assessment
‘For general evaluation, we can use the two dimensions, pleasantness and eventfulness. But we need more detailed information, especially for open-office areas’.	Additional soundscape descriptors		
‘Subjective factors, for me, are the most important because they shape our perception of the sound environment and also how we perceive the objective factors in terms of how much we are sensitive to them’.	Noise Sensitivity	Noise Sensitivity and Acoustic Adaptability	
‘. . . when we, of course, were looking into coping strategies, how people are actually behaving depending on this kind of sounds that they are hearing’.	Coping Strategies		

## Results

The results are structured based on the four guiding questions, each forming the basis for a separate thematic analysis. This approach allowed for a focussed exploration of themes relevant to each question while ensuring clarity in the presentation of findings. The themes identified for each question are presented below, along with supporting insights from expert discussions.

### Holistic approach to office soundscape assessment

**Figure 3.**
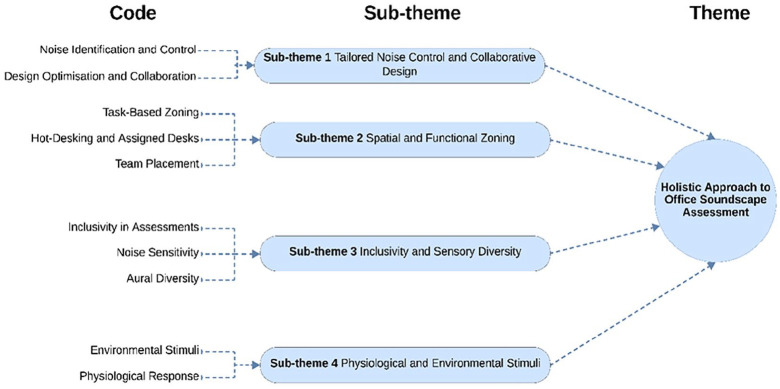
Exploring additional factors for office soundscape assessment.

The analysis of the first question identified four sub-themes, as shown in Figure 3: (1) Tailored Noise Control and Design Strategies, (2) Spatial and Functional Zoning, (3) Inclusivity and Sensory Diversity, (4) Physiological and Environmental Stimuli.

These sub-themes introduce new factors and offer deeper insights into existing ones, which should be considered in developing a soundscape assessment for open-plan office environments.

a. Tailored noise control and collaborative design

This sub-theme explores the critical role of noise control and design strategies in improving the soundscape quality of office environments. Experts highlighted the importance of identifying and managing noise sources, focussing on distinguishing between indoor and outdoor ones. As Participant #3 noted:

Identifying the origin, differentiating between indoor and outdoor noises, because it could require different control strategies.

This distinction is essential for implementing tailored noise control measures that address each source type’s specific characteristics and challenges, enhancing the overall acoustic environment.

Participant #3 also emphasised the need for design optimisation to refine the acoustic environment and assessment methods,

Design optimisation of indoor office environments and also the related assessment methods.

This highlights the importance of iterative improvements in office layouts and architectural planning, ensuring that interventions align with soundscape quality principles and user satisfaction.

Integrating stakeholders, such as consultants and estate teams, emerged as another factor in achieving effective design strategies. Experts pointed out potential challenges posed by misaligned priorities or poor communication. For instance, Participant #2 remarked:

Estates teams can significantly compromise the soundscape if (the) positioning is not aligned with acoustic goals.

This underscores the necessity of interdisciplinary collaboration among acoustic consultants, architects and facilities management to create environments that balance functionality and acoustic comfort.

b. Spatial and functional zoning

This sub-theme emerged as one of the factors considered in managing acoustic disturbances within open-plan offices. Experts highlighted the importance of task-based zoning, particularly in activity-based work settings. This approach allows users to perform tasks with varying noise tolerances by assigning specific zones to particular activities. As Participant #7 noted:

Zoning is only appropriate for activity-based working offices where you encourage people to behave differently in different places.

However, this strategy is less effective in environments with assigned desks, as the fixed nature of these workspaces limits the flexibility required for zoning to function effectively. Participant #7 further remarked:

For assigned desks, it’s meaningless to have zones for behaviour.

This suggests that spatial zoning is most effective in flexible workspace designs, emphasising the importance of aligning zoning strategies with the overall office layout.

Additionally, experts have also highlighted the detrimental impact of poor team placement within the workspace. For example, teams requiring high concentration levels are often placed near noisy areas, such as kitchens or IT help desks, leading to increased distractions. As Participant #2 noted:

Teams completing concentration tasks should not be positioned near kitchens where individual conversations are easily intelligible.

This indicates that even well-designed acoustic features may fail to mitigate the impact of poor spatial planning.

c. Inclusivity and sensory diversity

This sub-theme underscores the importance of inclusivity and sensory diversity in enhancing the comprehensiveness and equity of office soundscape assessments. Experts noted that traditional approaches to soundscape evaluations often fail to account for individuals’ diverse auditory and sensory needs, particularly those who are neurodivergent or have hearing loss. Participant #2 stated,

Making sure that any assessment method is inclusive and reflects people with lots of different needs, for example, neurodivergent or hearing loss.

This highlights the need for assessment methods that represent all user groups and address their unique needs. Experts further advocated for adopting more complex and nuanced approaches to assess noise sensitivity. Traditional binary questions, such as ‘I am sensitive to noise: Yes or No’, were criticised for oversimplification. As Participant #2 explained,

Rather than just a yes/no question on noise sensitivity, it needs to be more granular.

This approach allows for a richer understanding of individual responses to noise, enabling tailored interventions that align with diverse auditory experiences. Inclusivity also involves recognising that individuals’ auditory needs and sensitivities can vary not only due to physiological differences but also because of their roles and tasks within the workplace. For instance, a neurodivergent employee may require a quieter environment to maintain focus, while an individual with mild hearing loss may benefit from enhanced speech clarity in meeting areas. These diverse needs call for a multi-dimensional approach to soundscape assessment, ensuring that environments accommodate a broad spectrum of users.

d. Physiological and environmental stimuli

This sub-theme highlights the role of physiological and environmental stimuli in influencing acoustic perception in open-plan offices. Experts emphasised the interconnectedness of non-acoustic elements, such as thermal quality, air conditioning preferences and lighting, with users’ overall acoustic experiences. As Participant #6 noted,

Physiological response has a lot to do with the setting of the environment itself.

This highlights the role of environmental settings in shaping perceptions of soundscapes.

The discussion further revealed that individuals often prioritise certain environmental conditions before addressing acoustic quality. For example, preferences for thermal comfort or access to electrical chargers might precede sound-related factors. Participant #6 further described,

Perception of the thermal quality first. . . preferences for air conditioning at each desk or zone.

This suggests that users’ immediate physiological needs can influence their sensitivity and responses to acoustic environments.

These insights underscore the importance of adopting a multimodal approach in soundscape assessments. Rather than isolating acoustic factors, evaluations should account for how environmental stimuli interact with sound perception. For instance, poor lighting or uncomfortable thermal conditions could exacerbate perceived noise disturbances, even if the acoustic environment is objectively well-managed. The dynamic interaction between stimuli suggests that soundscape assessments should be designed to reflect real-world complexities.

These findings emphasise the need to go beyond traditional acoustic parameters by addressing additional factors identified by experts. Tailored noise control measures are essential for managing specific noise sources, while spatial and functional zoning ensures workspace layouts align with task requirements and minimise issues like poor team placement. Inclusivity and sensory diversity highlight the importance of assessments that accommodate diverse user needs, such as those of neurodivergent individuals and people with hearing impairments. Moreover, the interplay between physiological and environmental factors, including thermal comfort and lighting, underscores the need to integrate non-acoustic elements into soundscape evaluations. Collectively, these insights advocate for a ‘**Holistic Approach to Office Soundscape Assessment’**, providing a comprehensive framework to design more effective and inclusive office environments.

### Effective prioritisation of key factors in open-plan office soundscape assessment

**Figure 4.**
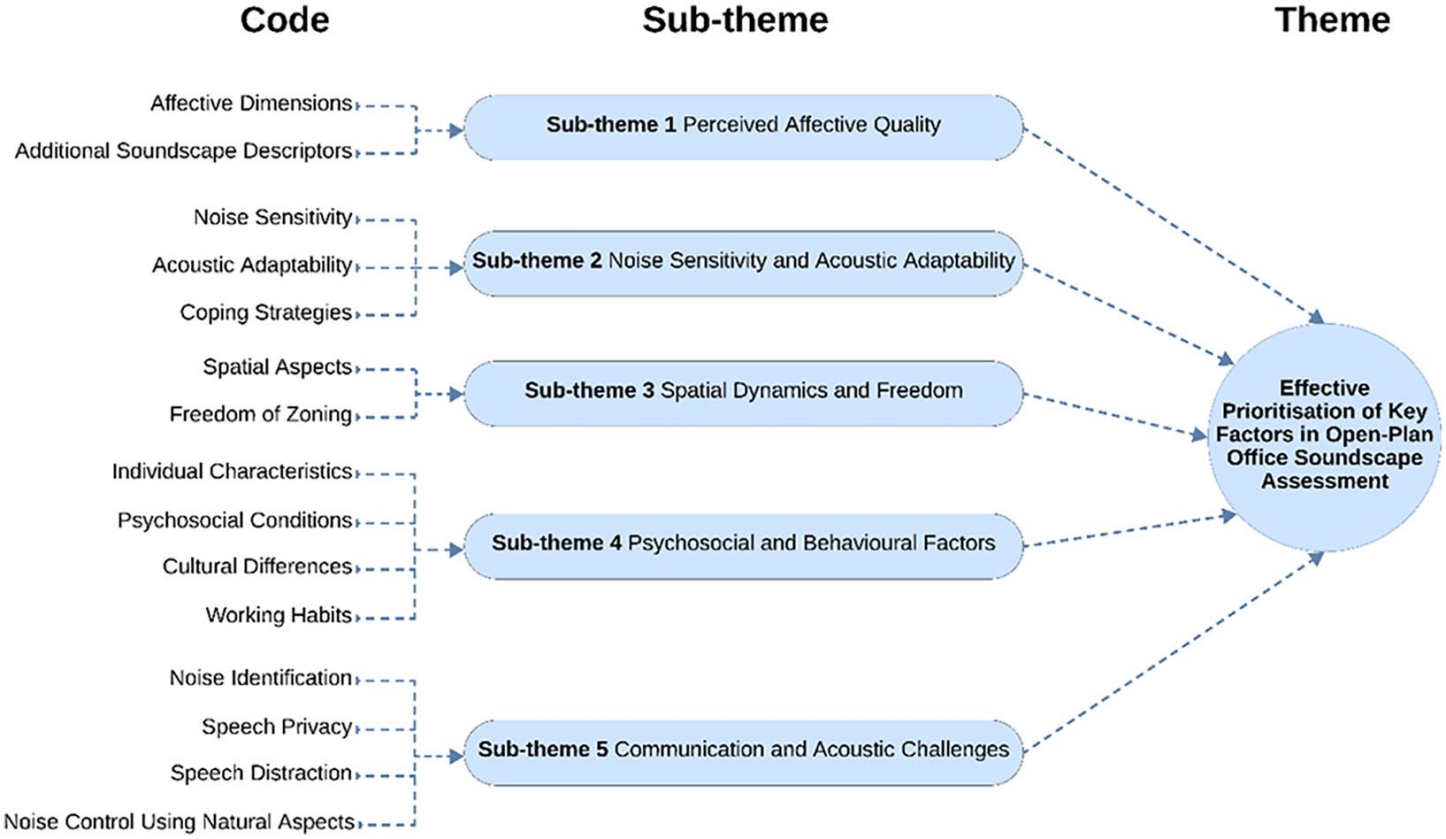
Prioritising key factors influencing open-plan office soundscape assessment.

The analysis for the second question resulted in five main themes, as illustrated in Figure 4: (1) Perceived Affective Quality, (2) Noise Sensitivity and Acoustic Adaptability, (3) Spatial Dynamics and Freedom, (4) Psychosocial and Behavioural Factors, (5) Communication and Acoustic Challenges. These sub-themes, derived from expert opinions and discussions, highlight important factors that should be prioritised in the soundscape assessment of Open-Plan Offices.

a. Perceived affective quality

This sub-theme examines how users of open-plan offices perceive soundscape quality and the factors influencing these perceptions. Expert discussions revealed that soundscapes influence users primarily through affective responses and contextual adaptations. Perceived Affective Qualities, such as pleasantness and eventfulness, outlined in ISO 12913, are central to soundscape assessment in open-plan offices. These dimensions capture emotional responses to acoustic environments and play a critical role in determining workplace comfort. As Participant #2 noted:

Pleasantness and eventfulness appear to be useful in open-plan office environments. But there are probably always some conceptual effects that are outside of those two dimensions.

Similarly, Participant #5 remarked:

For general evaluation, we can use the two dimensions, pleasantness and eventfulness. But we need more detailed information, especially for open-office areas.

While pleasantness and eventfulness provide a foundational framework for soundscape evaluation, experts emphasised the need for additional soundscape descriptors to capture their impact fully. For example, task-specific soundscapes were identified as crucial, as different tasks benefit from varying acoustic environments. Participant #5 explained:

The limited freedom is because we need to do specific tasks in specific areas, and we don’t have many options to do the same activity in different spaces.

This adaptability is reflected in the relationship between soundscape characteristics and task requirements. As Participant #2 noted:

Eventful soundscapes might be more productive for creative tasks, while calm soundscapes could better support tasks requiring high concentration.

Designing soundscapes for specific tasks can show positive benefits, such as eventful soundscapes being more productive for creative tasks.

These insights highlight the need for additional descriptors, such as supportiveness and task-specific alignment, to capture better how soundscapes facilitate goal achievement within a workspace. While pleasantness and eventfulness remain fundamental, incorporating dimensions like task adaptability and acoustic suitability offers a more nuanced understanding of soundscape quality.

b. Noise sensitivity and acoustic adaptability.

This sub-theme explores how individual differences in noise sensitivity and the adaptability of acoustic environments shape user experiences in open-plan offices. Discussions underscored the critical role of subjective factors, such as personal noise tolerance and coping strategies, in shaping how users perceive and interact with soundscapes.

Noise sensitivity, as a subjective trait, significantly influences users’ experiences of soundscapes in open-plan offices. It varies among individuals and is shaped by personal characteristics and work contexts. As Participant #3 explained:

Subjective factors, for me, are the most important because they shape our perception of the sound environment and also how we perceive the objective factors in terms of how much we are sensitive to them.

This highlights that noise sensitivity is not just a technical issue but also reflects individual perceptions of their work environment, including comfort, productivity and stress levels. Highly sensitive individuals are more affected by subtle sounds, such as conversations, footsteps or office equipment, which can disrupt tasks requiring deep focus. Participant #8 noted:

I think it’s very important what people are doing and how they are dealing with the sound at the time.

Noise sensitivity is dynamic and task-dependent. Activities requiring intense concentration heighten sensitivity to acoustic disturbances, while collaborative tasks are more tolerant of noise, provided it does not dominate the workspace.

Interestingly, experts also noted that overly quiet environments can exacerbate noise sensitivity. As Participant #7 and Participant #4 observed:

We get more complaints about people saying that it’s too noisy in offices when we would go and think it’s because it’s too quiet.

Louder contributing to the loudness of the sound, right? And if it is actually quieter there, you are even more significant contributing to that.

These observations suggest that excessively quiet settings may amplify minor sounds, causing discomfort. Balanced solutions, such as introducing soft background noise, can create a stable and comfortable acoustic environment for individuals with varying sensitivity levels.

Acoustic adaptability also emerged as a key consideration in soundscape assessments for open-plan offices. Participant #3 emphasised its importance within the broader framework of subjective factors:

. . . I placed firstly [prioritise], individual characteristics and working habits and then a physical condition, acoustic adaptability and noise sensitivity followed by the objective factors.

This statement underscores the interplay between subjective and objective factors, with acoustic adaptability enabling environments to meet diverse needs, whether supporting focussed tasks or facilitating collaborative activities.

Controlled ambient noise settings and individual coping strategies were identified as critical for managing noise in open-plan offices. Coping strategies, such as using headphones, help maintain productivity and comfort. However, Participant #6 and Participant #4 noted drawbacks:

When it’s too loud, people can develop some coping strategies like they can just put on earphones or headphones, but at the same time, when they are working, they cannot do that because they have to communicate with others in the same working area. So what happened is that there’s this speech privacy involved. . .

When the noise level is too high, people would prefer to have a very individual sound field or experience. They will use earphones, but that becomes a barrier when they need to communicate with others.

While headphones provide a personal acoustic zone, they can isolate users from social interactions and collaboration, creating barriers to communication when required in shared working areas. This highlights the importance of understanding coping strategies, as Participant #8 noted:

. . . when we, of course, were looking into coping strategies, how people are actually behaving depending on this kind of sounds that they are hearing.

This highlights the diversity in individual responses to noise and the need to tailor coping strategies to accommodate varying preferences. While some individuals may prefer to adapt to their environment, others rely on behavioural strategies, such as redirecting focus or tolerating noise to some degree. These mental adaptations form part of broader coping strategies, with individual traits and contextual factors influencing tolerance levels.

c. Spatial dynamics and freedom

This sub-theme explores how spatial configurations in open-plan offices influence users’ sense of autonomy and satisfaction. Experts emphasised that while such spaces are intended to promote flexibility, constraints related to mobility and layout can limit this experience in practice. Across the discussion, participants highlighted the importance of providing diverse zones, clear adjacency between functions, and flexible seating arrangements to accommodate varying needs and preferences.

One of the topics raised in the discussion was the importance of the ability to choose where and how one works within the space. Participants noted that having limited freedom to move or select a workspace could significantly influence satisfaction. As Participant #7 explained:

They chose to sit at [a noisy area near the meeting room], but they were free to move and decided they preferred sitting there and putting up with the sound.

This statement underscores how personal choice, even in suboptimal conditions, can foster a sense of agency. Participant #7 further noted:

The way you feel about your environment in terms of what’s important to how you perceive it really starts with the sense of control that you have over finding the environment that you need, and that suits you.

Conversely, a lack of control, such as being assigned a specific desk or working in a constrained space, can reduce satisfaction and productivity. Expanding on these insights, hot desking, where employees lack fixed seating, introduces additional complexity to spatial dynamics. Participant #1 noted that this flexibility creates unique opportunities for soundscape assessment, such as soundwalks:

In an office with hot desking, the Soundwalk methodology could be employed because people are not working statically. They are moving. . . . It would be a way to administer a survey and take the measurements.

Soundwalks offer a dynamic way to assess spatial experiences, capturing movement and fluctuating perceptions throughout the office. Participants also emphasised that spatial quality is defined by more than flexibility. Participant #7 remarked:

I don’t think we can characterise a space as being good. It’s about how people interact with it and use it in different contexts.

Clearly defined zones within the office enabled task-specific functionality and enhanced spatial usability. As Participant #5 explained:

We need to map different zones in the office and evaluate their suitability for various tasks. Each zone should offer a distinct function to support diverse activities.

These insights underscore the need for intentional design strategies that match spatial functions with the specific demands of daily work activities. Participants expressed a strong desire for autonomy in choosing where to work based on their tasks. However, they also noted limitations in the available space and flexibility that restricted such choices. Participant #5 noted:

The freedom for doing different activities in the same space is very limited in the open office. We don’t have many places to move to, and everyone is moving, but you don’t have enough space to be in your own little area.

Although open-plan offices are designed to support mobility and flexibility, participants described how spatial constraints often prevent users from relocating to areas that better suit their tasks or comfort. Participant #5 further added:

In urban areas, we can move to different places for different activities. But in open offices, tasks often dictate where you must stay, limiting your freedom to choose more positive spaces.

This lack of adaptability affects not only productivity but also overall satisfaction. As Participant #1 observed:

Everyone is moving, but you don’t have space to move somewhere else and you end up stuck in tiny spaces.

In addition, conflicting spatial and acoustic needs frequently led to dissatisfaction. For instance, participants described situations where team-based activities requiring collaborative efforts occurred near individuals performing detail-oriented work. Participant #7 noted:

You get the conflicts where you get some people who want to talk [and] other people who don’t.

These tensions reveal the difficulty of accommodating contrasting spatial and acoustic preferences within a shared environment. Addressing such challenges requires thoughtful design strategies, including the provision of quiet zones for individual tasks and designated collaborative areas for group discussions to ensure that different working styles coexist. However, achieving this balance presents practical challenges. As Participant #1 noted:

Planning and architectural layout, and we need space and that’s money for managing buildings or building [new ones].

This underscores the resource trade-offs involved in creating flexible and inclusive office environments. Organisations must balance budgetary constraints with designing spaces that effectively accommodate diverse user preferences.

d. Psychosocial and behavioural factors

This sub-theme explores how psychosocial and behavioural factors influence experiences in open-plan offices. Expert discussions revealed the complex interplay between individual characteristics, psychological conditions, cultural differences and working habits that shape how employees perceive and adapt to their environments, impacting well-being and productivity.

**Individual characteristics**, encompassing personality traits and behaviours, significantly influence how employees interact with open-plan office environments. Participants emphasised that these characteristics vary widely, influencing perceptions and responses to the surrounding environment. They identified this as a key factor to consider, as reflected in the Mentimeter responses shared by four experts. This behavioural variability highlights the need to incorporate psychological or psychosocial factors into soundscape assessments. As Participant #8 noted:

I think so, because I mean, we’re all different and I think the way we experience these factors (psychological aspects) is also different.

Participant #8 further elaborated:

I think these psychological and also psychosocial aspects are very relevant for sound experience because they tell me a lot about how people [perceive and interact with the environment].

Some employees adapt well to shared spaces, finding them stimulating and collaborative, while others experience discomfort or frustration. For instance, extroverted individuals may thrive in collaborative areas, whereas introverts often prefer quieter, more secluded environments.

Psychological conditions, such as anxiety and self-consciousness, also shape experiences in open-plan offices. Participant #4 observed:

Some people are just anxious, thinking they might disturb others or be overheard, which significantly affects their mood and productivity.

Feelings of discomfort can be exacerbated by the open layout, underscoring the need to design environments that reduce stress and foster a sense of control.

In addition to psychological conditions, cultural backgrounds further shape responses to soundscapes in shared environments. Participant #6 highlighted how cultural norms influence tolerance towards noise:

In some cultures, people talk a lot and they don’t really care about involuntary listeners from what they are discussing, but in some, that is something very private or considered kind of annoying.

Cultural differences contribute to variability in how individuals perceive and navigate open-plan offices. For instance, some cultures may normalise open communication and tolerate noise, while others prioritise privacy and find noise disruptive. As Participant #6 further elaborated:

The acceptance about noise level is different, including the speech privacy in some cultures.

Working habits, including task execution and strategies for navigating open-plan office environments, are shaped by both individual and cultural factors. These habits reflect personality traits as well as broader cultural and social contexts. For instance, individuals from collectivist cultures may gravitate towards collaborative areas, while those from individualist cultures may prefer quieter zones for independent tasks.

The interaction of individual, psychological and cultural factors highlights the importance of designing flexible office spaces. By incorporating distinct zones for collaborative and focussed work, open-plan offices can better accommodate diverse work styles and preferences, supporting a multicultural workforce and fostering inclusivity and productivity.

e. Communication and acoustic challenges

This sub-theme explores the communication and acoustic challenges in open-plan offices, focussing on noise identification, speech privacy, speech distraction and noise control using natural resources or ambience and their impact on workplace experiences.

Identifying and classifying noise is fundamental to managing soundscapes in open-plan offices. Experts highlighted the need to refine sound source categorisation to suit indoor office environments. Participant #2 remarked:

. . .For the other questions, there’s probably some tweaks required, particularly to the sound source types that obviously wouldn’t be appropriate in indoors at all, but certainly not in offices.

This highlights the need to adapt soundscape evaluation frameworks, such as those from ISO 12913, to account for the unique characteristics of open-plan offices. Participant #1 emphasised the relationship between sound dominance and the refinement of source categorisation:

. . .we need to refine maybe the type of sources we are considering when assessing some dominance.

Distinguishing dominant sounds, such as conversations or machinery, from ambient background noise is particularly challenging in complex acoustic environments. Contextual factors—like proximity, user activity and spatial layout—also play a role. For example, users may tolerate specific noise sources if they have control over their seating arrangements, underscoring the need for categorisation methods that reflect both physical and perceptual aspects of noise.

Speech privacy emerged as another critical concern in open-plan offices, where intelligible conversations can lead to involuntary listening and compromised confidentiality. Participant #6 noted:

Speech privacy is particularly important because if you don’t have it, people feel they can’t focus or they feel self-conscious about talking.

Insufficient speech privacy disrupts workplace dynamics and increases stress. Participant #6 further explained the role of voluntary and involuntary listeners in shaping privacy experiences:

So what happened is that there’s this speech privacy involves, people that are actually voluntary listeners and then people that are involuntary listeners. They get to condition the privacy is for the involuntary hearing, and the other **one** is the speech distraction or working distraction.

This observation reveals the dual challenges of managing conversations to protect speech privacy while minimising distractions for unintended listeners. Acoustic design strategies, such as sound insulation, are key in addressing these issues. As Participant #4 expert suggested:

Yes, [noise insulation metrics] can be included to add information about the characteristics of the space.

By integrating such metrics, workplaces can better align room characteristics with the diverse acoustic needs of their users. Participant #4 further noted that this perspective aligns with the response from Participant #7 on Mentimeter, emphasising that user perception is strongly influenced by their sense of control over the environment. This highlights how sound insulation supports privacy and empowers users to choose environments best suited to their tasks. For example, highly insulated spaces may be ideal for confidential conversations or focussed work, while open, less insulated areas can foster collaborative activities.

However, participants also noted that achieving an optimal acoustic environment involves more than managing insulation levels. Interestingly, excessively quiet environments can also heighten anxiety. Participant #4 observed:

The problem is mostly not with the office environment being too loud, but with it being too quiet. When it’s quieter, some people feel more nervous, thinking they are being overheard or might disturb others.

To address this issue, participants suggested introducing low-level ambient sounds, such as natural soundscapes, to balance the acoustic environment. Natural sounds, like birdsongs and water features, can create a calming and pleasant acoustic environment, enhancing workplace productivity and well-being. Participant #3 explained:

When people were exposed to bird sound, even if they could not see the birds, we found a positive correlation with cognitive test results.

The alignment of auditory cues with visual elements was also emphasised to avoid perceptual dissonance. For example, a water sound should be paired with a visible water feature to prevent confusion or discomfort. As Participant #8 stated:

If you want to put like a water sound, we need also to put at least like an ornament of the water in the office.

Masking intrusive office noise with natural soundscapes is another effective strategy, though its success depends on the appropriateness of the sounds to the environment. Generic natural soundtracks may not always seamlessly integrate into an office setting. As Participant #8 further explained:

The sounds should make sense. For example, if you hear water and there’s a water ornament, then you know it’s the waterfall in the room making the sound. But if you only hear water, it just makes you need to go to the toilet.

Incorporating biophilic design principles—such as natural sounds and complementary visual features—can create a more harmonious and restorative work environment. These elements not only mitigate noise disturbances but also enhance the aesthetic and functional quality of the workspace, contributing to employee well-being and productivity.

These findings suggest that elements such as perceived affective quality, noise sensitivity, spatial dynamics, psychosocial factors and communication challenges should be considered in open-plan office soundscape evaluations. Combining these factors makes the assessment more effective as it addresses the complex interactions between the acoustic environment and various factors influencing individual experiences. For example, by prioritising individual characteristics such as noise sensitivity and coping strategies, the evaluation can better reflect how individuals perceive and adapt to acoustic discomfort in open-plan offices. Based on these findings, they propose the **‘Effective Prioritisation of Key Factors in Open-Plan Office Soundscape Assessment,’** which combines these elements to create a more adaptive soundscape assessment that aligns with the real-world complexities of open-plan office offices, providing a more comprehensive and applicable understanding.

### The harmony between subjective and objective factors

**Figure 5.**
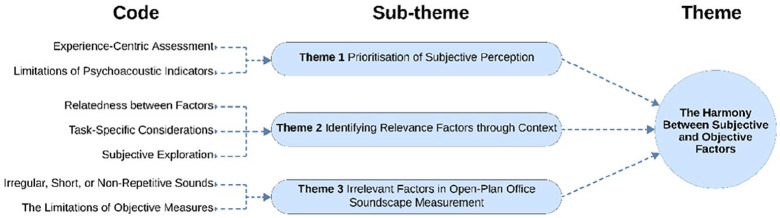
Relevance and exclusion of certain acoustic and non-acoustic factors.

This analysis identified three key themes, as shown in Figure 5: (1) Prioritisation of Subjective Perception, (2) Identifying Relevance Factors through Context, and (3) Irrelevant Factors in Open-Plan Office Soundscape Assessment.

a. Prioritisation of subjective perception

This sub-theme underscores the centrality of subjective user perceptions in soundscape assessment for open-plan offices, with physical measurements serving a supporting role. The discussions revealed interconnected aspects between the need for experience-centric assessments and the limitations of psychoacoustic or objective indicators.

Experts emphasised that soundscape assessments must prioritise user experiences, as subjective responses such as pleasantness and comfort offer deeper insights into workplace dynamics than objective metrics. As Participant #2 explained:

The perception of the people using a particular space is more important.

Participant #7 also added:

We need to understand how well people like an office and why they like it. . . the experience needs to come first.

This perspective shifts the focus of soundscape assessments towards understanding how acoustic environments support workplace activities. Participant #2 reinforced this idea:

Measuring the perception of those people is probably a lot more important than measuring physical characteristics.

While physical measurements—such as psychoacoustic indicators, noise levels and reverberation time—provide measurable data, experts agreed that they should complement, not replace, subjective evaluations. As Participant #2 pointed out, their limitations:

Room and psychoacoustic indicators [are] being unreliable . . .

Participant #7 elaborated:

The measures can only support the experience. . . I don’t think you can infer the experience from measuring anything.

These reflections highlight that psychoacoustic indicators, while helpful, cannot fully explain how users experience soundscapes. Instead, they should validate and support subjective findings, ensuring a comprehensive understanding of the acoustic environment’s impact on workplace satisfaction.

b. Identifying relevance factors through context

This sub-theme explores the importance of contextual relevance in identifying acoustic and non-acoustic factors for soundscape assessments in open-plan offices. Experts emphasised that the interrelation of factors, task-specific considerations and subjective exploration are critical to determining which elements should be included.

Experts noted that many objective and subjective factors are strongly interrelated, requiring a strategic approach to identify the most impactful ones. Rather than including all potential factors, assessments should focus on those with the greatest contextual importance. As Participant #8 noted:

You have, of course, all kinds of things that you mention also in the objective and subjective that might be strongly related, but also cognitive task, cognitive load, work performance. All these kinds of aspects seem a bit related to me.

This underscores the need to streamline assessments by avoiding redundancy. Participant #8 further suggested:

See which ones are kind of talking about the same thing, and then pick **one** of those because a lot of these things, of course, have to do with each other.

By understanding the overlap between related factors, the assessment can focus on the most relevant and distinct contributors, ensuring clarity and efficiency. As Participant #6 explained:

For most important factors . . . try not to, you know, categorise, which is good or bad, but try to find which factor is the dominant factor to really dig in what you call the people perception. . .

This finding highlights that the focus shifts to understanding which elements most meaningfully shape the acoustic experience in specific contexts rather than rigid categorisation. Such an approach ensures that soundscape assessments remain targeted and relevant, effectively addressing user needs.

The relevance of factors also depends on their alignment with task requirements. Experts highlighted that specific tasks or user groups may necessitate tailored soundscape indicators or factors. As Participant #2 explained:

One office with a particular set of indicators [factors] could work for **one** group and not for another group. . .

This insight reflects the need to adapt soundscape assessments to specific tasks and user groups, as the same environment may be perceived differently depending on the nature of work or the individuals involved. Integrating task relevance ensures that evaluations align with diverse workplace activities, such as communication, concentration or collaboration.

Experts also emphasised the importance of engaging participants directly to identify relevant factors through subjective exploration. Methods like semi-structured interviews uncover more profound insights into why occupants feel a certain way about their workspace and which factors they consider significant. As Participant #4 suggested:

We should first focus on identifying why people feel the way they do to that sort of place and then look at what that place actually includes and what it sounds like. Is it appropriate?

This approach prioritises subjective insights, ensuring that assessments capture the acoustic characteristics of the space and its perceived appropriateness. By emphasising subjective exploration, soundscape evaluations can identify contextually relevant factors that might be overlooked in purely objective analyses.

c. Irrelevance factors in open-plan office soundscape assessment

This sub-theme examines specific factors deemed irrelevant to soundscape assessments for open-plan offices, focussing on three main areas: irregular, short or non-repetitive sounds and the limitations of objective measures. These factors were identified as having minimal influence on user perception or failing to capture the broader soundscape experience.

Experts noted that irregular, short, or non-repetitive sounds—such as sudden, isolated noises—do not reflect the consistent acoustic patterns typical of office environments. While noticeable, these transient sounds were considered to have limited relevance for long-term soundscape perception. As Participant #3 explained:

Like sudden or short noises, like **one** shot. I mean. . . sudden noises are not typical of an office environment. It can be. . . not as much as much disruptive head noise, for instance. Or a repetitive sound.

These insights suggest that irregular and one-off sounds do not contribute meaningfully to soundscape assessments, as they lack the consistent influence required to shape users’ perceptions over time. Similarly, temporary and atypical sounds—such as one-off noises from screens or other devices—were considered irrelevant. These noises are fleeting and uncommon, failing to represent a typical office’s stable and predictable conditions. Participant #3 further remarked:

From an input level point of view, I’m not talking about sounds from HVAC, but for instance from. . . I don’t know a screen, which is like a one-shot. . .

The participant further explained that these atypical noises are unlikely to disrupt overall perceptions of the soundscape:

It is not typical of my usual sound environment and which not to disrupt my perception of the soundscape because it is not a repetitive sound.

Temporary and atypical sounds are situational and inconsistent, making them unsuitable for inclusion in assessments focussed on long-term or recurring soundscape experiences.

In addition to identifying irrelevant sounds, experts highlighted the limitations of traditional objective measures, such as noise levels, reverberation time and sound distribution metrics. While these parameters provide quantifiable data, they are insufficient for capturing the users’ subjective experience. Participant #2 remarked:

Measuring things like noise levels or quantifying the space with reverberation time, destruction distance, all those parameters. . . it’s, I suppose, we’re kind of all really tempted to try and find what the combination of those things is that results in good perceptual response.

Participant #4 further elaborated:

The physical measurements do not reflect the office environment very well.

While supportive, these insights emphasise that objective parameters cannot fully explain user perceptions or experiences. Their inability to address contextual and emotional factors limits their standalone effectiveness in soundscape evaluations.

These findings emphasise the importance of a nuanced approach to soundscape assessment in open-plan offices, prioritising subjective user experiences to understand better how acoustic environments influence workplace dynamics. Excluding irrelevant factors, such as non-representative sounds and limiting the reliance on objective measures make the evaluation process more focussed and efficient. These results highlight the critical need to prioritise subjective insights while eliminating unnecessary elements, leading to the proposed framework: **‘The Harmony Between Subjective and Objective Factors.’** This framework ensures a balanced integration of user-centred evaluations and empirical data, enabling a comprehensive and practical assessment of soundscapes in open-plan offices.

### Tailoring soundscape questionnaires to contextual and practical needs

**Figure 6.**
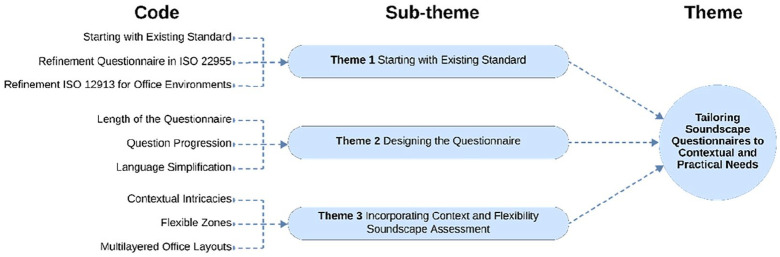
Integration of key factors into soundscape questionnaire design.

The analysis for the fourth question led to the identification of three main themes as illustrated in Figure 6: (1) Starting with the existing standard, (2) Designing the questionnaire, (3) Incorporating Context and Flexibility in Soundscape Assessment.

a. Starting with existing standards

This sub-theme explores the application of established ISO standards as a foundation for designing soundscape assessment tools in office environments, highlighting their strengths while identifying areas for refinement. Expert discussions underscored the relevance of ISO 12913 and ISO 22955 as foundational references while highlighting the need for contextual and task-specific adaptations.

ISO 12913, initially developed for outdoor soundscapes, provides a practical conceptual framework for assessing acoustic environments. Experts emphasised its utility in structuring initial assessments for indoor spaces, as Participant #2 noted:

The questionnaire being similar to ISO 12913 is useful. . . the two-dimensional model would still be useful for identifying those issues.

Similarly, ISO 22955 offers practical guidelines for open-plan office design, particularly in categorising task types and their acoustic requirements. As Participant #2 further observed:

I use the ISO 22955 task types in the questionnaire because that was a starting point.

However, the discussions revealed certain limitations when applying these standards to the nuanced dynamics of office environments. The expert emphasised the need to refine task categories and contextual descriptors to better align with user experiences:

These are the task types. . . but they probably need some refinement (ISO 22955). The wording of those categories may be what led to some uncertainty in the responses.

Additionally, Participant #2 noted that ISO 12913 may not fully account for contextual variations specific to indoor environments:

There’s a lot of contextual information that it doesn’t collect, particularly for office environments.

These insights highlight the need to tailor existing standards to address the unique requirements of indoor workspaces. While ISO 12913 and ISO 22955 provide foundational structures, enhancing these standards to include more explicit task definitions, demographic considerations and contextual adaptations can improve their applicability to office soundscapes.

b. Designing the questionnaire

This sub-theme explores key considerations for structuring soundscape questionnaires in office environments, focussing on length, question progression and language simplification. Expert discussions highlighted the influence of these elements on participant engagement and response quality, emphasising the need to balance practicality with comprehensiveness.

The length of the questionnaire, in terms of both time and number of questions, emerged as a critical concern. Completion time significantly affects participants’ willingness to engage, particularly in workplaces. Participant #8 reflected on the challenges of a lengthy survey:

We have a survey at the moment that takes on average 35 minutes in the public sector, and we have 10,000 respondents. Some people are obviously willing. . . but I think personally it’s too long.

This underscores the importance of designing questionnaires with manageable durations to avoid discouraging participation, especially in busy office environments.

Experts further emphasised limiting the number of questions to sustain participants’ attention, particularly in practical evaluations involving office workers. As Participant #1 remarked:

If it’s a starting point where we are paying participants, that’s okay. . . but for a practical evaluation with normal workers, it’s way too long for their attention and time to dedicate to a passive survey.

This suggests that while longer questionnaires may be acceptable in research settings where participants are compensated, practical workplace surveys should focus on essential questions to respect participants’ time and engagement.

Another key recommendation was to begin the questionnaire with general questions and then move towards more specific ones. This structure allows participants to engage with the survey more comfortably, minimising the risk of feeling overwhelmed. As Participant #6 explained:

I’d capture first the perception that goes with something more general and then you go to a little bit more detail with the factors.

Finally, simplifying questionnaire language was identified as a crucial factor for improving participant comprehension and data reliability. Experts noted that technical terminology often intended for practitioners could confuse participants, as noted by Participant #2:

Those phrases are worded for the practitioners. . . not necessarily the participant group of a survey.

When asked whether clearer instructions or simpler language were necessary to improve the questionnaire’s usability, Participant #2 responded:

Yeah, probably. Simpler language. In the questionnaire, I guess.

Moreover, to enhance participant understanding, Participant #6 suggested using tailored descriptors written in everyday language:

I would have a different scale. . . tailored descriptors would be a good choice.

This highlights that simplifying language improves clarity and ensures that questions resonate with participants, resulting in more accurate and reliable responses.

c. Incorporating context and flexibility in soundscape assessment

This sub-theme examines how acoustic and non-acoustic factors, such as contextual intricacies, flexible zones and office layouts, can be effectively integrated into the design of a soundscape questionnaire. Expert discussions highlighted the importance of capturing the diversity of tasks, user needs and environmental dynamics within office spaces.

Experts highlighted the value of including questions that gather contextual and demographic information to understand better the factors influencing soundscape perceptions. Collecting such data enables researchers to identify the reasons behind positive or negative ratings and better understand how to respond to users’ needs. Participant #2 noted:

And that’s not the design that we were trying to achieve and then try and find out why from perhaps some of the more contextual information that could be collected with the survey.

This underscores the significance of demographic details—such as age, role or department—alongside activity-specific data in providing deeper insights into how individuals interact with their acoustic environment, Participant #2 further added:

What are the people doing in that space. . . demographic type information helps understand why a soundscape has been rated as annoying or pleasant.

An interesting perspective raised by Participant #5 linked economic satisfaction, a non-acoustic factor, to how people perceive their acoustic environment:

If people get happy with the payment that they get, they will be really happy with the environment.

Including questions on such aspects ensures that the questionnaire comprehensively captures user experiences across diverse contexts, enabling soundscape designs that align with varying needs and expectations.

Questions about flexible zones were deemed essential for understanding the role of spatial arrangements in shaping soundscape perceptions. Participant #4 stressed the importance of assessing the impact of quiet zones, defensible spaces and task-specific areas:

You should be able to have this defensible space where you can just work in peace and quiet without getting any distractions.

This suggests that a soundscape questionnaire should evaluate the availability and effectiveness of such zones, along with their impact on employee comfort and productivity.

Another key consideration was a multi-layered approach to office layouts, where areas are tailored to the acoustic needs of specific tasks. Participant #4 further recommended including questions that assess how well office layouts meet task requirements:

Louder departments go towards the louder part of the office. . . but there should be flexibility to move to quieter zones as needed.

Exploring employees’ perceptions of these layered layouts can help identify areas for improvement and ensure office designs better align with user needs.

The findings suggest that soundscape questionnaires should be tailored to the dynamics of open-plan offices by incorporating both acoustic and non-acoustic factors. While the existing standards provide a solid foundation, their application must be refined to encompass the full range of activities, experiences and contextual factors relevant to open-plan offices. Practical considerations, including ensuring manageable questionnaire lengths, using simplified language and structuring questions progressively, were identified as critical for maintaining participant engagement and enhancing response reliability. These insights underscore the importance of **‘Tailoring Soundscape Questionnaires to Contextual and Practical Needs,’** offering a framework that reflects real-world office environments’ complexities and diverse demands.

## Discussions

### Additional factors in office soundscape assessment

This section discusses potential new factors proposed by experts during the thematic analysis. The goal is to critically evaluate and compare these factors with those previously identified in the systematic review. By integrating expert insights with established findings, this analysis aims to identify additional dimensions that could enhance the framework for soundscape assessment in open-plan offices.

a. Noise sources and control strategies

The thematic analysis emphasised the distinction between indoor and outdoor noise sources, consistent with the findings of the systematic review.^27^ However, studies included in the review indicated that indoor noise is typically the dominant source of acoustic disturbances. Outdoor noise sources were grouped under the category ‘Other Background Noise,’ encompassing sounds such as human activity, equipment operation, traffic noise and construction noise.^49–53^ The analysis emphasised the importance of tailored control strategies for each type of noise source to improve noise management. This aligns with the systematic review, which identified questionnaires addressing passive, active and personal noise control strategies.^27^

Passive strategies focussed on mitigating deficiencies in sound insulation in windows, walls and floors, identified as significant contributors to acoustic dissatisfaction.^53^ Studies involving questionnaires on privacy and workspace separation were also discussed in the review. These studies assessed the effectiveness of walls, panels and furnishings in providing seclusion, supporting concentration and reducing distractions.^54,55^ Additionally, dedicated spaces for specific activities, such as online meetings or phone calls, were examined for their contribution to privacy and functionality in office environments.^56^ These findings underscore the need for improved soundproofing materials workspace separation to enhance noise control and acoustic privacy.

Active strategies, on the other hand, introduced solutions like sound masking systems to mitigate noise disturbances.^57–64^ Studies evaluated the effectiveness of these systems, including preferences for natural masking sounds, such as water features,^57^ and the adequacy of noise masking levels.^62^ Several studies assessed the effectiveness of these systems by evaluating the adequacy of noise masking levels^62^ and explored participants’ preferences for maintaining the use of water features as a permanent masking system in office environments.^57^ While sound masking can create acoustically balanced workplaces, experts cautioned about unintended consequences. For instance, a study found that water features increased toilet visits, indicating broader impacts on physical comfort and daily routines.^57^

Personal strategies involved tools like headphones or earplugs, widely used to isolate individuals from noise distractions.^20,51,55^ Studies assessed occupant satisfaction with these measures, particularly their effectiveness in reducing noise disturbances and enhancing overall acoustic quality.

b. Spatial and functional design in acoustic management

The thematic analysis also highlighted the critical role of the estate team in ensuring successful acoustic design. An expert emphasised that errors in the placement of design elements by this team could compromise acoustic objectives. This issue is categorised as an objective factor, as it involves technical decisions in spatial design. Although strategic stakeholder involvement in the design process was not a primary focus, the systematic review successfully identified multiple studies investigating spatial design strategies.^27^ These studies focussed on analysing spatial layouts and their impact on employees’ work experiences. For example, some approaches combined evaluations of office layouts through architectural drawings and direct observations to identify diverse work zones, such as quiet areas, semi-quiet zones and collaborative spaces. These studies also assessed how design elements supported visual and acoustic privacy.^21,54^ Observational methods further examined employees’ adaptation to office environments and their interaction patterns with the spatial design. These observations were systematically documented, providing insights into how office design influences work experiences and employees’ responses to disruptions.^20^

To complement the objective approach, the systematic review highlighted the use of questionnaires to evaluate design strategies within the context of spatial dynamics.^27^ These questionnaires examined factors like the degree of enclosure, the distance between employees and the adequacy of personal workspace, which significantly impact user satisfaction.^63^ Similarly, other studies explored the adequacy of personal and visitor spaces, as well as the impact of proximity to colleagues on overall comfort and functionality.^52,55^ Workstation positioning emerged as a critical element, with studies examining its effects on user experiences. For instance, research investigated the influence of proximity to windows or doors and the placement of workstations.^21,52,57,65^ These findings underscore the importance of spatial configurations in shaping workplace dynamics and enhancing employee satisfaction.

Thematic analysis further identified spatial and functional zoning as a key strategy for managing acoustic disturbances, particularly in activity-based flexible office environments. This approach allocates zones based on noise tolerance levels. However, an expert highlighted that its effectiveness is limited to office types with activity-based designs, as demonstrated in studies in the systematic review.^54,55^ Details of questionnaire items related to zoning strategies will be discussed in the next section.

c. Inclusivity in soundscape assessment

The thematic analysis also emphasised the importance of inclusive assessment methods that address diverse auditory needs, particularly for neurodivergent individuals or those with hearing impairments. This aligns with the review’s findings that current soundscape assessments often rely on standardised tools, overlooking individual variability.^27^ This underscores the urgent need for more inclusive frameworks beyond generalised approaches.

Furthermore, the systematic review identified various noise sensitivity tools, such as the NoiseQ Sensitivity and Weinstein’s Noise-Sensitivity Scale, alongside binary noise sensitivity questions and adapted versions of existing tools.^21,32,57–59,61,64–68^ While these tools measure differences in noise sensitivity among individuals, they often lack the depth needed to ensure inclusivity and to fully address the needs of diverse groups, including individuals with disabilities.

For instance, individuals with Autism Spectrum Disorder (ASD) often experience sensory sensitivities across multiple modalities, including auditory, visual, tactile, olfactory and gustatory systems.^69–72^ Auditory challenges, such as heightened sensitivity to background noise, can disrupt concentration and speech perception more significantly in individuals with ASD than in typically developed individuals.^72^ Rosas-Pérez et al.^73^ explored these auditory experiences in educational environments through semi-structured online interviews with 12 autistic adults (aged 25–64). Participants were asked to reflect on sounds or situations that negatively impacted their well-being, those that had positive effects, common challenges, coping strategies, and potential improvement.^73^ The study underscores the need for soundscape assessments for individuals with disabilities, advocating for a holistic approach that integrates emotional responses, personal experiences and contextual factors into the evaluation process. It also suggests that current research on acoustics and perception in the general population may overlook common challenges faced by individuals with disabilities.

d. Environmental and physiological influences on acoustic perception

Thematic analysis has provided new insights into the influence of physiological and environmental stimuli, building upon the findings of the previous systematic review.^27^ Experts noted that thermal quality, such as the individualised adjustment of air conditioning at each workstation or zone, is often prioritised over acoustic aspects. This suggests that suboptimal environmental conditions, such as uncomfortable temperatures, can exacerbate perceptions of noise disturbances, even in acoustically well-managed environments. Although some studies in the systematic review addressed the relationship between environmental stimuli and acoustic perception,^24,52,53,63,74–76^ the primary focus was on soundscape perception,^27^ which limited the identification of environmental stimuli as influencing factors.

Several studies explored various aspects of comfort and environmental perception in workspaces, encompassing thermal, visual, psychological and acoustic dimensions, utilising diverse approaches and survey instruments.^24,52,53,63,74–76^ Thermal environments were evaluated by assessing thermal comfort parameters such as temperature, humidity and thermal preferences, employing standards like ANSI/ASHRAE Standard 55 and ISO 10551, as well as examining the effects of noise on thermal perception.^24,52,63,74,76^ Visual environments were assessed based on lighting and room aesthetics,^52,53,63,75^ while indoor air quality was evaluated through perceptions of air quality and its impact on comfort.^52,63,75,76^

In the systematic review, various physiological parameters were employed to assess bodily responses to environmental stimuli, office conditions and cognitive tasks, focussing on stress, emotional states and physiological arousal. These parameters included blood pressure, heart rate, oxygen saturation, respiratory rate, pleth variability index, perfusion index, electrodermal activity, and skin temperature.^23,77^ The review also discussed questionnaires as complementary tools for measuring physical conditions. Self-perception of health was assessed using questionnaires and the WHO’s Health at Work survey.^21,57,58,65,66^ Fatigue and exhaustion were evaluated using instruments such as the Checklist Individual Strength and the Multidimensional Fatigue Inventory.^21,58,66^ Pain, including back pain, neck pain, headaches, eye strain, and vertigo, was also analysed.^21,24,57,65,66^ Sleep quality and alertness were assessed using tools such as the PSQ, the WHO’s Health at Work survey and the modified Karolinska Sleepiness Scale.^59–61,78^ These measures offer valuable insights into the impact of office environments on physical well-being.

e. Socio-cultural and economic factors

The thematic analysis revealed additional factors to be considered in soundscape assessment in open-plan offices. Although not identified in the discussion for the first question, this factor emerged in responses to other questions, making it relevant to categorise it as an additional factor. One expert emphasised the importance of cultural background in shaping individuals’ responses to soundscapes, particularly in terms of noise tolerance and speech privacy. Some cultures prefer noise and open communication, while others prioritise silence and privacy. Work habits are also shaped by cultural values, with collectivist cultures favouring collaborative spaces and individualist cultures preferring quieter areas. Other soundscape studies have discussed the significance of social-cultural characteristics, though these studies did not primarily focus on open-plan offices.^79–82^ This factor was previously highlighted in a study by Indrani et al.,^21^ as summarised in the systematic review.^27^ However, the questionnaire content regarding ‘social-cultural characteristics’ overlapped with the concept of acoustic adaptability, which evaluates individuals’ ability to adjust to noise, including their tolerance for disturbances caused by colleagues.^27^

Another aspect the thematic analysis identifies is the potential inclusion of economic factors, such as salary satisfaction, in soundscape assessment for open-plan offices. One expert suggested that satisfaction with income is strongly linked to overall job satisfaction,^83,84^ which often positively influences the work experience. Job satisfaction was previously categorised as a work performance factor in the systematic review.^27^

The studies in the systematic review employed various approaches to measure job satisfaction. Some studies used general questions about overall job satisfaction,^62^ while others adopted three out of six items from the Global Job Satisfaction (GJS) scale,^67^ which includes Job Decision Satisfaction, Recommendation Satisfaction, Ideal Job Comparison, Expectations Satisfaction, Overall Job Satisfaction and Job Liking.^85^ Additionally, Specific Job Satisfaction facets, including salary satisfaction, were assessed to provide a more comprehensive understanding of job satisfaction.^85^

These studies highlighted that while job and salary satisfaction are interrelated, they are measured using separate scales to capture their unique dimensions. This approach enables a more nuanced analysis of the relationship between job satisfaction and economic factors, providing comprehensive insights into the role of economic aspects in shaping workplace experiences, particularly in open-plan offices.

### Prioritisation and relevance of factors in open-plan office soundscape assessment

The thematic analysis underscores the critical role of subjective user perceptions in soundscape assessments for open-plan offices. Experts assert that soundscape evaluations should prioritise user experiences, as subjective responses offer more profound insights into workplace dynamics than objective metrics. Additionally, identifying essential factors for inclusion in soundscape assessments is crucial for ensuring a holistic and context-sensitive understanding of user experiences. This section will discuss the factors considered essential to prioritise.

a. Perceived affective quality

The analysis emphasises the importance of understanding how users of open-plan offices perceive soundscape quality and the factors influencing these perceptions. Experts highlighted affective dimensions, such as pleasantness and eventfulness—adapted from ISO 12913—as key elements in evaluating soundscape quality.^86^ While these dimensions are foundational, experts suggest that more specific descriptors are required to address diverse workplace needs. Moreover, task-specific dimensions should be integrated into assessments to support various activities in such environments.

A separate study on soundscape assessments in open-plan offices identified three perceptual dimensions: pleasantness, eventfulness and a novel dimension termed emptiness. The concept of emptiness captures a sense of detachment, even in the presence of distant background sounds, highlighting the disconnect between physical and acoustic presence, often observed in offices with low occupancy levels.^25^ Complementing these findings, the systematic review identified a range of descriptors that expand the understanding of soundscape characteristics in office settings.^27^ Terms like calm, tranquil and peaceful evoke a sense of tranquillity,^21,23,24,29,87^ while pleasantness highlights comfort and positivity.^23,24,29,57,59–62,68^ Negative impacts, such as noise disturbances, are expressed as distracting, annoying, noise annoyance, stressful, distress and nervous.^22,23,29,62,74,88–90^ Dynamic qualities emerge in descriptors like lively, exciting, energetic, active, and vibrant,^21–23,29,87^ contrasting with boring or monotonous perceptions.^23,24,29^ Physical sound properties are also highlighted, from loudness^18,90,91^ to chaotic or turbulent complexity.^23,24,29,87^ Specific acoustic features, such as reverberant, rumble, roar and hiss, add texture to evaluations.^90,92^

Despite considerable efforts in identifying descriptors and dimensions, integrating task-relevant dimensions requires further investigation. Additional studies exploring correlations between emerging dimensions and specific task types are essential to ensure the relevance of soundscapes to workplace activities. By bridging these gaps, future research can advance the design of soundscapes that effectively support diverse work environments and enhance user experiences.

b. Individual differences and noise management strategies

The thematic analysis further highlights that individual differences in noise sensitivity significantly impact soundscape experiences. Noise sensitivity, a subjective trait, varies among individuals and is closely tied to task demands. Experts note that individuals with high sensitivity are more susceptible to disturbances from subtle sounds, while tasks requiring lower concentration can tolerate higher noise levels. These findings align with studies showing that individuals with high noise sensitivity experience greater disturbance, lower acoustic satisfaction, more difficulty adapting, higher subjective workloads and reduced performance compared to those with low sensitivity.^32,59,64,65,67,68,93^ Measurement tools previously discussed offer valuable insights into this variability.

Acoustic adaptability emerges as another critical factor in soundscape assessments for open-plan offices. The ability to adjust the acoustic environment allows workspaces to accommodate the diverse needs of users. This systematic review explores questionnaire content related to acoustic adaptability, offering additional insights for a more holistic soundscape evaluation.^27^ These questionnaires assess participants’ capacity to adapt to acoustic conditions. Some evaluate general adaptability to acoustic environments,^32,62^ while others examine adaptation to the surroundings of a sound environment.^59–61,68^ Other studies address noise adaptation in offices, including tolerance of ambient sounds or coworkers’ behaviour, as well as habituation to noise levels.^21^ Another aspect measured is participants’ willingness to listen to various types of sounds during work hours.^77^

Subsequently, experts suggest that controlled ambient sounds or natural soundscapes effectively balance acoustic environments, fostering concentration and collaboration. Sound masking systems have been shown to improve workplace acoustics, enhancing productivity and tranquillity while accommodating individual preferences through flexible designs.^22^ For instance, certain configurations, like spring water sounds at medium SNR (−2.4 dB), can boost task performance, speech privacy and acoustic satisfaction while reducing disturbances. However, improper settings may increase cognitive workload.^64^ Water-based masking sounds offer potential benefits but require careful sound spectrum design and alignment with user needs.^62^ While these systems enhance acoustic comfort and focus, their effectiveness in improving speech privacy is limited in smaller office settings.^57^ Conventional sound masking systems can reduce speech intelligibility but may inadvertently amplify distractions from other noise sources, highlighting the need for integrated, holistic approaches.^66^ Adaptive sound masking systems further contribute by reducing distractions, enhancing well-being and lowering reliance on coping strategies, ultimately improving workplace engagement and mental health.^58^ These findings collectively emphasise the importance of thoughtful sound masking design in creating balanced, productive, open-plan office environments.

Experts also emphasise that coping strategies, such as using headphones, can help maintain productivity and comfort. However, they caution against excessive reliance on headphones, which may isolate users from social interactions. This concern aligns with a study showing that while headphones can enhance concentration, they also hinder communication.^20^ To address this, the systematic review identifies various alternative coping strategies that can be integrated into soundscape assessments for open-plan offices, offering a more comprehensive framework to support user well-being in the workplace.^27^

Coping strategies for managing workplace noise can be divided into active and passive approaches. Active strategies involve deliberate actions to address noise disturbances. These include using noise-cancelling tools such as headphones, earphones, or earplugs to play music or block out disruptive sounds.^20,51,55^ Other actions involve temporarily leaving the workspace, relocating to quieter areas or working remotely.^20,51,58,88^ Communication-based strategies, such as discussing noise concerns with colleagues, adjusting work rhythms or asking coworkers to lower their voices, are also common.^20,51,58,88^ Additionally, workers might manage noise levels proactively by speaking softly, using designated spaces for private conversations, or stepping outside for phone calls.^20,51,58^ Reporting noise issues to management or suggesting improvements to the acoustic environment further demonstrates active engagement in noise management.^51,58,88^

Passive strategies, on the other hand, focus on adapting to the noise rather than altering it. These include gradually acclimating to the existing noise levels or choosing to ignore disruptive sounds.^51^ Some workers adopt a focus-driven mindset, exerting extra effort to complete tasks despite unfavourable acoustic conditions.^51^ Such strategies reflect an adaptive approach, where the individual prioritises endurance over direct environmental modification.

This classification of coping strategies provides valuable insights into how office users cope with noise, offering a foundation for more comprehensive, and user-centred soundscape assessment approaches.

c. Spatial configurations and flexibility

The thematic analysis also emphasises the importance of spatial configurations and the perceived freedom to move within open-plan offices in shaping user experiences. The ability to choose one’s workspace fosters a sense of control and satisfaction. Experts highlight the value of creating task-specific zones, such as quiet areas for focussed tasks and collaborative zones for discussions. This approach is commonly supported by activity-based flexible offices (AFOs), which allow users to select non-assigned workspaces based on their activities or preferences.^94^ However, insufficient zone diversity in AFOs can lead to reduced productivity, increased stress, overcrowding, confusion over zone usage, feelings of isolation and dissatisfaction with individual and social work environments.^54^ A clear and balanced division of zones is therefore essential to support diverse activities, including tasks requiring concentration and collaboration. The systematic review further identified questionnaire content related to spatial configurations, categorised under the broader factor of space dynamics.^27^

Regarding spatial configurations, the questionnaire items include analyses of the physical location of workstations, such as proximity to windows or doors.^21,57,65^ These items also compare office layouts—such as cellular offices, shared spaces and open-plan designs—to understand cognitive perceptions of the office environment.^78^ Other items explore space utilisation and preferences, investigating how individuals use available space, preferences for specific zones and usage frequency.^21,29^ Additionally, space dynamics factors include questionnaire items that assess satisfaction with privacy and workspace separation.^54,55^

d. Behavioural, psychological and cultural factors

The analysis further explores the influence of behavioural, psychosocial and cultural factors on soundscape perceptions in open-plan offices. Experts discuss the critical role of individual characteristics, such as personality traits and behaviours, in shaping interactions and perceptions of the work environment. Evidence indicates that introverts and individuals with high neuroticism are more adversely affected by noise, reporting significant declines in productivity, concentration and overall well-being. Similarly, lower levels of conscientiousness and agreeableness are linked to increased stress, distraction and conversational interference. Coping strategies also vary, with extroverts demonstrating greater noise tolerance, while individuals with high neuroticism often adopt avoidance-based responses. These findings underscore the necessity of integrating personality-informed considerations into soundscape design to address the diverse needs of users.^50,51^ According to the systematic review, the Big Five Inventory (BFI) is commonly used to assess individual characteristics.^27^ This tool measures the five major dimensions of human personality—Openness, Conscientiousness, Extraversion, Agreeableness, and Neuroticism—collectively known as the OCEAN model.^95–97^ The BFI, developed through many studies,^95–98^ is available in various versions, including a full 44-item version and a shorter 10-item version for ease of use.^27^ Some studies focus on specific personality dimensions, linking them to particular behaviours, such as social preferences or interpersonal relationships in specific contexts.^21,51,61^

Similarly, experts emphasise that psychological conditions, such as anxiety or self-consciousness, can significantly impact individual mood and productivity, making them critical considerations in workplace design. Several validated tools identified through the systematic review offer structured and reliable approaches to assess these conditions. For instance, the PHQ-4 (Four-Item Patient Health Questionnaire for Anxiety and Depression) evaluates symptoms of anxiety and depression by addressing feelings of nervousness, difficulty controlling worry, sadness or hopelessness and loss of interest or pleasure in daily activities.^58,99^ Additionally, the Oldenburg Burnout Inventory (OLBI), as applied by the same authors, assesses work-related exhaustion and engagement by measuring dimensions of exhaustion and disengagement.^100^

Other tools, such as the UWIST Mood Adjective Checklist, provide insights into individuals’ emotional states at work by evaluating Hedonic Tone and Tense Arousal.^58,101^ Meanwhile, the Multidimensional Fatigue Inventory (MFI-20) examines the effects of fatigue on motivation and work performance.^66^ Collectively, these instruments offer systematic and validated methodologies for assessing psychological conditions within office environments.

For the social interactions factor, various studies included in the systematic review have utilised developed or modified surveys to evaluate workplace social interactions within the context of soundscapes. These surveys assess aspects such as noise levels conducive to communication, creating a dynamic work atmosphere, team collaboration, and employee engagement. They also address relational conflicts, including disagreements and non-work-related issues.^20,54,56^

On the other hand, a study used a psychosocial questionnaire to investigate how moving into an activity-based workplace (ABW) impacts satisfaction with communication, social relations (including social support and a sense of community) and work demands (quantitative, emotional and work pace).^102^ This questionnaire was adapted from various related studies.^103–106^

Cultural norms are also highlighted as an important factor for inclusion in soundscape assessments for open-plan offices. This factor has been discussed in detail in a previous section, underscoring its relevance in creating work environments that cater to diverse user needs.

e. Less relevant factors and holistic integration

The thematic analysis explores less relevant factors for soundscape assessments in open-plan offices. One expert highlighted that irrelevant sounds, such as irregular and non-repetitive sounds, have minimal impact on long-term soundscape perception. This aspect relates to sound source identification, where experts suggest revising corresponding questions in the ISO 12913 soundscape assessment questionnaire.^107^ Details of these revisions will be elaborated in a subsequent section. Moreover, experts argue that the relevance of factors depends on the assessment’s specific focus. It is unnecessary to include factors with overlapping objectives; instead, selecting the most representative factor ensures alignment with the soundscape assessment’s goals, maintaining a balanced approach.

Experts further emphasise that while reliable and quantifiable, traditional objective measurements, such as noise levels, reverberation time, and sound distribution, are insufficient for capturing the subjective dimensions of user experience. Although these metrics provide insights into the technical aspects of the acoustic environment, they fail to account for contextual and emotional factors that shape user perceptions. This aligns with research indicating that conventional acoustic metrics are inadequate for addressing the full range of factors influencing user satisfaction in open-plan offices. Integrating subjective insights with objective data, a soundscape approach offers a more holistic understanding of the acoustic environment and ensures designs address both technical and human needs.^108^ Subsequently, experts recommend conducting objective measurements after collecting subjective data. This sequencing allows for correlating technical measurements with user experiences, enabling a more holistic understanding of acoustic environment quality in open-plan offices.

### Important factors to consider in soundscape questionnaire design

The thematic analysis of expert input provides valuable insights for designing soundscape questionnaires tailored to office environments. This section discusses the key factors and approaches to be implemented in the questionnaire design.

a. Leveraging existing standards for questionnaire frameworks

Experts highlighted the importance of leveraging existing ISO standards, such as ISO 12913 and Annexe D of ISO 22955, as foundational frameworks.^31,91^ These standards offer structured approaches for assessing acoustic environments, providing conceptual models and practical guidelines for preliminary evaluations of office spaces. However, discussions revealed limitations in applying these standards to the complex dynamics of office environments. For instance, ISO 12913, initially designed for outdoor soundscapes, requires contextual adaptations to address specific office factors, such as sound source identification. The systematic review successfully identified relevant sound sources in office settings, categorised into three groups: mechanical equipment and machine, human activities, other background noise.^27^ Machines contribute sounds from systems such as ventilation, air conditioning, lighting and office equipment like computers, photocopiers and telephones.^19,21,24,49–52,57,65,66,88^ Human activities encompass conversations (both in-person, monologue and over the phone), footsteps, laughter and interactions with furniture.^19,21,49–53,56,57,65,66^ Other background noise includes external sounds, such as traffic and construction, as well as indoor sources, like radios and kitchen appliances.^19,49–53^ Similarly, the task classifications outlined in ISO 22955 require refinement to address the intricacies of contemporary office activities. A recent study found no statistically significant correlation between task type and soundscape perception scores, emphasising the need for a more nuanced framework for task-specific soundscape preferences.^25^

b. Balancing questionnaire length and participant engagement

Experts also stressed the importance of balancing comprehensiveness with participant engagement. Questionnaire length, measured by completion time and the number of questions, is critical. Overly lengthy questionnaires risk reduced participation rates, especially in busy work environments. Studies show that survey fatigue increases significantly with longer surveys, with each additional hour of completion time raising the likelihood of skipped questions by 10%–64%.^109^ Furthermore, the negative effects of excessive questionnaire length have been extensively documented.^110,111^ For example, one expert cited that a 35-min survey, despite achieving high response rates, was deemed excessively long by participants. The median acceptable duration for online surveys is 15 min, with respondents’ attention spans averaging around 20 min.^112^ To maintain engagement, studies recommend designing questionnaires with 25–30 questions, taking no more than 30 min to complete.^113^ Experts suggested structuring questionnaires to minimise respondent fatigue and improve data quality, starting with general questions and gradually progressing to more specific ones. This approach allows participants to engage without feeling overwhelmed and is widely supported in the literature.^112^ In such cases, questionnaire designers should be prepared to explain the rationale for transitions.

c. Language accessibility and clarity

Using clear and straightforward language is fundamental to effective questionnaire design. Experts noted that technical terms, such as those in the Annexe D of ISO 22955,^114^ may confuse participants, especially when targeting individuals with varying familiarity with soundscape concepts. Replacing jargon with plain, relatable language aligned with participants’ daily experiences enhances reliability and engagement. This aligns with literature emphasising that questionnaires should be clear, unambiguous and devoid of technical or inappropriate language to improve reliability.^115,116^ For instance, one study used simple, experience-based language for response options, recognising that respondents often have limited knowledge of acoustics.^117^

Overall, effective soundscape questionnaires require a balance between practicality for participants and comprehensiveness in capturing relevant data. This involves prioritising essential questions aligned with the study’s objectives, avoiding excessive detail and ensuring accessibility. Simplified language, logical question flow and thoughtful consideration of length collectively ensure that the questionnaire remains accessible, engaging and effective in collecting meaningful insights.

### Developing effective soundscape assessment tools using experts’ Insights

This section focuses on enhancing soundscape assessment tools by incorporating comprehensive input from the focus group discussions. The goal is to develop a more effective, inclusive and representative tool that captures the diverse phenomena occurring in open-plan offices.

Experts emphasised the importance of using existing standards, such as ISO 12913, as a foundational framework for soundscape assessment. ISO 12913 provides robust methodologies and detailed evaluation guidelines. However, the experts highlighted the need for modifications and contextual adjustments to address open-plan offices’ unique challenges and characteristics. Consequently, expert discussions led to the selection of both acoustic and non-acoustic factors to be incorporated into the assessment.

a. Individual characteristics

- **Noise Sensitivity:** The reduced version of the NoiseQ Sensitivity questionnaire (NoiseQ-R Sensitivity), developed by Griefahn, can be used to evaluate noise sensitivity across three key domains: sleep, habitation, and work.^118^ The NoiseQ-R Sensitivity is a 12-item questionnaire and has been previously applied in the GABO Questionnaire (Annexe D of ISO 22955).^114^ For a more concise alternative, the Short Form of the Weinstein Noise Sensitivity Scale (NSS-SF) is available. This version retains the key measurement properties of the original scale and consists of five items rated on a 6-point Likert scale.^119–122^
- **Sample statements:**
  ○ Sleep: I do not feel well-rested after a noisy night.
  ○ Habitation: I am very sensitive to noise from my neighbours.
  ○ Work: I need peace and quiet to perform a difficult task.
-  **Personality traits:** The Big Five Inventory (BFI) is a widely used psychological assessment tool designed to measure the Big Five personality traits: Openness to Experience, Conscientiousness, Extraversion, Agreeableness and Neuroticism. Alternatively, the shorter version of the BFI, consisting of 10 statements using a 5-point Likert scale and taking less than a minute to complete,^98^ can be integrated into this assessment.
-  **Sample questions:**
  ○ Openness to Experience: I see myself as someone who has an active imagination.
  ○ Conscientiousness: I see myself as someone who does a thorough job.
  ○ Extraversion: I see myself as someone who is outgoing and sociable.
  ○ Agreeableness: I see myself as someone who is generally trusting.
  ○ Neuroticism: I see myself as someone who gets nervous easily.
-  **Disability and special needs:** The question regarding Disability and Special Needs was taken from the Survey on Diversity, Inclusion and Respect at the Workplace conducted by the European Commission.^123^ Consequently, it is essential to implement additional or specialised approaches—adaptive methods, accessible tools, or tailored communication strategies—to ensure that individuals with disabilities and special needs can actively participate in soundscape assessments in open-plan offices.
-  **Sample questions:**
  ○ Do you have a disability?

- *Disability refers to long-term physical, mental, intellectual or sensory impairments that may limit full participation in society.*
-  Yes/No/No, but I have a temporary impairment/Prefer not to say
  ○ If yes, please indicate the type of disability (you can choose more than one):
     Physical disability/Intellectual or sensory impairment/Visual impairment/Hearing impairment/Psychosocial disability/Other/Prefer not to say.

**b. Sound Source Identification:** Questions regarding sound source identification can be adapted from ISO 12913-2.^107^ However, the list of sound sources needs further adjustment to reflect the specific conditions of open-plan offices. The sound source categories have been discussed in detail in Sections 4.1 and 4.2.

**Sample Questions:**
 How often do you hear the following noises at your workstation? Please rate each on a scale from 1 (Never) to 5 (Constantly).
 List of Noise: (Include the adjusted list of sound sources here, tailored to open-plan office environments.)

**c. Perceived Affective Quality:** Questions related to perceived affective quality can be adapted from ISO 12913-2. However, based on expert discussions, additional descriptors are needed to reflect better real situations in open-plan offices, such as descriptors that represent task-specific dimensions. Furthermore, a recent study identified a new dimension in open-plan offices, referred to as emptiness.^25^ Further investigation is required to integrate task-relevant dimensions into the assessment framework effectively.

**Sample Questions:**
 For each of the following characteristics, please indicate the extent to which you agree or disagree that the current surrounding sound environment is:
• Pleasant
• Chaotic
• Vibrant
 . . .

**d. Coping Strategies:** For questions regarding coping strategies, the options for coping strategies are derived from studies included in the systematic review.^27^ Detailed explanations have been provided in Section 4.2.

**Sample Questions:**
 For each of the strategies listed below, please indicate how often you use them to cope with noise in your workplace.
• Use earphones or headphones
• Discuss the noise issue with colleagues
• Tried to be quieter in hopes others would do the same
 . . .

**e. Job Satisfaction**: Questions related to job satisfaction can be adapted from the study by Park et al., which utilised the Global Job Satisfaction questionnaire.^67^ This scale consists of six items rated on a 5-point Likert scale and was designed to capture an employee’s overall satisfaction with their job. As an alternative, the Short Index of Job Satisfaction (SIJS) can be considered. This shorter version includes five items, rated on a 5-point Likert scale and provides an efficient yet reliable measure of overall job satisfaction.^124–126^

**Sample Questions:**
• Enthusiasm about my work: In general, how much do you like your job?
• Enjoyment in my work: How does this job compare to your ideal job?
• Satisfaction with my present job: All things considered, how satisfied are you with your current job?

**f. Psychosocial:** The psychosocial aspects were derived from the study by Haapakangas et al., which included questions about satisfaction with communication, social support from colleagues, social community at work and work demands.^102^

**Sample Questions:**
• Satisfaction with communication: How satisfied are you with information exchange with your closest colleagues (on work-related subjects)?
• Social support from colleagues: How often do your colleagues talk with you about how well you carry out your work?
• Social community at work: Do you feel part of a community at your place of work?
• Quantitative Demands: Do you have enough time for your work tasks?
• Emotional Demands: Does your work put you in emotionally disturbing situations?
• Work Pace: Do you work at a high pace throughout the day?

**g. Space Dynamics:** Questions related to space dynamics or spatial configurations can be adapted from studies included in the systematic review.^27^ Detailed information regarding these questions has been discussed in Section 4.2.

**Sample Questions:**
• **How would you rate the density of your office space in terms of:**
  ○ The degree of enclosure of your work area by walls, screens or furniture.
  ○ The distance between you and your colleagues.
  ○ The adequacy of your personal workspace for accommodating work, materials and visitors.
• **Which area do you prefer for the following tasks? (Select one for each task)**
Focussed work/Collaborative work/Informal discussions/Taking breaks
  ○ Workstation
  ○ Meeting room
  ○ Quiet zone
  ○ Other (please specify): ___________

The factors presented above, which integrate acoustic and non-acoustic, were carefully considered to avoid overlaps and ensure clarity in their categorisation. Nonetheless, some of these factors may be reconsidered, and additional ones may be introduced to improve the clarity and applicability of the assessment. This paper also includes a sample questionnaire for assessing soundscapes in open-plan offices, which was developed based on the outcomes of the expert discussions. The complete questionnaire is available in the Supplemental Materials.

**Figure 7.**
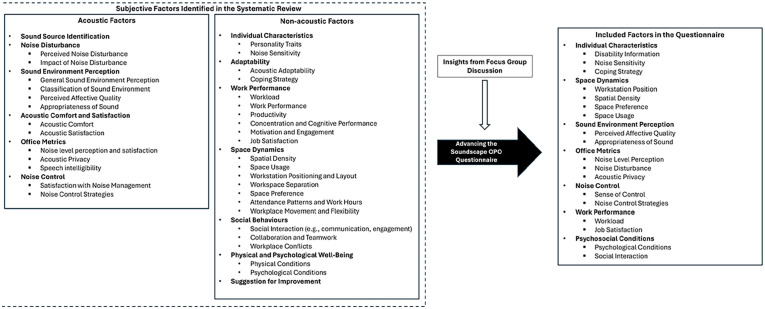
Advancement of assessment factors using systematic review and expert input.

Figure 7 illustrates how insights from expert discussions contributed to refining the factors identified through the systematic review^27^ and incorporating newly proposed factors. Developing this assessment tool requires iterative testing and refinement to ensure it is effective and practical across diverse settings. The tool’s effectiveness should be evaluated to determine its ability to capture critical elements of soundscape perception. Furthermore, scales must be carefully designed to produce accurate and interpretable data. Ease of completion is another critical aspect, with the questionnaire designed to be user-friendly and accessible to participants regardless of their background. Lastly, ensuring clarity of the questions through simple, non-technical language is essential to enhance the reliability and validity of the assessment results.

## Limitations

The use of qualitative methods, such as focus group discussions and thematic analysis, may inherently reflect the researchers’ perspectives and interpretations. The research team, comprising three experts in acoustics and soundscapes—Z.R. (Indonesian, fluent in Indonesian and English), F.A. (Italian, fluent in Italian and English), and J.K. (Chinese, fluent in Mandarin and English)—acknowledges that their disciplinary focus and cultural perspectives may have shaped the framing of research questions and interpretation of findings. Efforts to minimise bias included validating findings through group consensus and integrating diverse perspectives. Additionally, the study was limited by selecting a small group of experts specialising in building acoustics, soundscape studies and workplace studies. Input from broader disciplines, such as cognitive sciences, organisational behaviour and user-centred design, would further enrich the findings. Moreover, time constraints also restricted the duration of focus group sessions, which may have limited the opportunity to explore additional perspectives and insights. Although a structured protocol was applied, as detailed in the Materials and Methods section, future studies may benefit from conducting multiple rounds to allow for richer exploration. Additionally, conducting follow-up interviews may help capture deeper individual perspectives that might not fully emerge in a group setting.

## Conclusions

This paper successfully conducted focus group discussions with experts to advance soundscape assessment methodologies for open-plan office environments, providing a more comprehensive and practical framework alongside actionable tools, such as questionnaires, for researchers, designers, and policymakers.

To address the first research question, the thematic analysis identified several additional factors to enhance soundscape assessments in open-plan offices. These include developing inclusive frameworks that consider the auditory needs of individuals with disabilities, such as neurodivergent and hard-of-hearing individuals, to address diverse sensitivities adequately. Environmental stimuli can be considered an additional factor, particularly if the aim is to explore the relationship between acoustic stimuli and other environmental elements. Cultural background was also highlighted as a key determinant that shapes noise tolerance, communication privacy, and work style preferences, although these aspects often intersect with other influencing factors. Additionally, economic aspects, such as salary satisfaction, warrant further exploration due to their potential impact on job satisfaction. For objective assessments, additional factors include engaging estate teams in the acoustic spatial design process to ensure effective solutions for diverse activities within open-plan offices.

Regarding the second research question, the thematic analysis revealed that subjective user perceptions heavily influence soundscape quality in open-plan offices. Affective dimensions, such as pleasantness and eventfulness (as defined in ISO 12913), are fundamental to soundscape evaluation. However, new dimensions are necessary to capture the variety of activities in open-plan offices. Individual differences—noise sensitivity, acoustic adaptability, personality traits and behavioural, psychosocial and cultural factors—are critical for understanding users’ ability to manage disturbances and their overall soundscape perception. Experts recommended incorporating coping strategies into assessments to gain deeper insights into how users address challenges within these dynamic environments.

Spatial configurations and perceived freedom of movement within the office also play a crucial role in shaping soundscape experiences, particularly in activity-based work environments that promote mobility. Experts highlighted integrating sound masking systems as an effective intervention, provided these systems are carefully designed to enhance the soundscape experience. While not inherently subjective, sound masking systems can be included in questionnaires to evaluate the effectiveness of active design strategies.

Depending on the context, some factors were deemed less critical for soundscape assessments in open-plan offices. For example, irregular and non-repetitive sounds were found to have minimal long-term impact on perception and can be excluded unless assessments focus on transient disruptions. This study adjusted the types of sounds included in assessments accordingly. Experts also recommended simplifying overlapping factors to ensure clarity and ease of understanding for participants. Objective measures, such as noise levels and reverberation times, were also considered insufficient to capture subjective user experiences. These measures should complement soundscape approaches to provide a holistic understanding of the acoustic environment.

Addressing the third research question, integrating key factors into soundscape assessment tools like questionnaires can leverage ISO 12913 and ISO 22955 as foundational frameworks. These frameworks should be adapted to reflect office-specific dynamics, such as sound source categorisation and task classification. To balance participant engagement with data quality, questionnaires should remain concise (25–30 questions within 30 min), maintain a logical flow and prioritise key questions. Clear, accessible language devoid of technical jargon enhances reliability and inclusivity. These approaches enable soundscape assessments to capture meaningful insights while remaining practical and user-centred.

In response to the fourth research question, expert contributions provide valuable guidance for shaping effective soundscape assessment tools tailored to open-plan offices. By building on existing standards as a foundational framework, adjustments can be made to address the specific challenges of these environments. Through an iterative design process that emphasises clarity, inclusivity and practicality, the tools can be refined to capture the complexities of office soundscapes effectively. These approaches ensure that the tools remain adaptable and user-centred while providing meaningful insights into the soundscape experiences of diverse open-plan offices.

In conclusion, this study advances soundscape assessment methodologies for open-plan office environments by addressing gaps in existing practices. By integrating acoustic and non-acoustic factors, accommodating diverse user needs and developing a detailed framework for practical tools such as questionnaires, this research provides a user-centred approach to evaluating office soundscapes. The framework lays the groundwork for designing effective assessment tools based on existing standards. Moving forward, an iterative design process that emphasises clarity, inclusivity and practicality is necessary to test and refine these tools, ensuring they effectively capture the complexities of office soundscapes. These contributions provide actionable insights for researchers, designers and policymakers to enhance acoustic environments, ultimately supporting employee well-being, productivity and overall satisfaction in modern office settings.

## Supplemental Material

sj-pdf-1-bua-10.1177_1351010X251340905 – Supplemental material for Advancing soundscape assessment in open-plan offices: Insights from expert focus groups Supplemental material, sj-pdf-1-bua-10.1177_1351010X251340905 for Advancing soundscape assessment in open-plan offices: Insights from expert focus groups by Zulfi Rachman, Francesco Aletta and Jian Kang in Building Acoustics
