# Supplementary material for: Advancing soundscape assessment in open-plan offices: Insights from expert focus groups
Source: Build Acoust. 2025 Jun 13;32(3):417–61. doi: 10.1177/1351010X251340905 (PMC13086230; doi:10.1177/1351010X251340905)
Supplement: sj-pdf-1-bua-10.1177_1351010X251340905 – Supplemental material for Advancing soundscape assessment in open-plan offices: Insights from expert focus groups [file sj-pdf-1-bua-10.1177_1351010X251340905.pdf]

Part I – Understanding You & Your Work Environment

This section of the Soundscape Assessment Questionnaire for Open-Plan Offices collects essential background information to better understand how individual differences and workplace conditions influence noise perception and soundscape experience.

A. Demographic Information - Please provide the following information.

1. **Gender:**
   ☐ Man ☐ Woman ☐ Non-binary ☐ Prefer not to say Specify, if you wish ________
2. **Age:**
   ☐18-24 ☐25-34 ☐35-44 ☐45-54 ☐55-64 ☐65+
3. **Years in the company or institution:**
   ☐ < 1 year ☐ 1 to 5 years ☐ > 5 years
4. **Do you have any conditions or experiences that affect how you perceive and interact with the sound environment?** (e.g., sensory hypersensitivity, misophonia, hyperacusis, hearing differences, auditory processing difficulties, neurodivergent conditions such as autism or ADHD). ***Optional***
   ☐ Yes, I'm happy to specify ____________________ ☐ Yes, but I prefer not to specify
   ☐ No ☐ Prefer not to say

B. General Information About Your Workstation

Please describe your workstation setup, workspace density, and access to private spaces in your office.

1. **Do you have a fixed workstation in your office?**
   ☐ Yes ☐ No
2. **How would you describe the workspace density?**
   ☐ Very crowded ☐ Somewhat crowded ☐ Moderate ☐ Spacious ☐ Very spacious
3. **How satisfied are you with the acoustic privacy provided by walls, panels, or furniture around your workstation?**
   ☐ 1 -Very dissatisfied ☐ 2 ☐ 3 ☐ 4 ☐ 5 – Very Satisfied

C. Your Sensitivity to Noise *- Please rate how much you agree with each statement.*

Below are several statements about noise sensitivity in different situations.

Statement	1 – Disagree Strongly	2	3	4	5	6 – Agree Strongly
I am sensitive to noise.	☐	☐	☐	☐	☐	☐
I get annoyed when my neighbours are noisy.	☐	☐	☐	☐	☐	☐
I get used to most noises without much difficulty. (R)	☐	☐	☐	☐	☐	☐
I find it hard to relax in a noisy environment.	☐	☐	☐	☐	☐	☐
I get frustrated when noise prevents me from falling asleep or completing my work.	☐	☐	☐	☐	☐	☐

D. Your Job Satisfaction

Please indicate how much you agree or disagree with each statement about your job.

Statement	1 – Strongly Disagree	2	3	4	5 – Strongly Agree
I feel fairly satisfied with my present job.	☐	☐	☐	☐	☐
Most days, I am enthusiastic about my work.	☐	☐	☐	☐	☐
Each day at work seems like it will never end. (R)	☐	☐	☐	☐	☐
I find real enjoyment in my work.	☐	☐	☐	☐	☐
I consider my job to be rather unpleasant. (R)	☐	☐	☐	☐	☐

DRAFT

Part II – Evaluating Your Office Sound Environment

This section examines how noise and the overall sound environment affect your work experience. Your responses will help us assess the relationship between office noise, employee well-being, and productivity.

A. Assessment Area - Please specify the office area you are evaluating in this questionnaire.

1. **Which area of the office are you evaluating?**
   Building Name:_____ Floor Number:_____ Area of the office: __________
2. **How frequently do you work in this area?**
   ☐ Daily ☐ Several times a week ☐ Occasionally ☐ Rarely

B. Workplace Noise and Disturbances - Please assess the noise conditions in your workplace by rating the overall noise level, the frequency of different noises, and the specific noises that you find most disturbing.

1. **Noise Level:** How would you rate the overall noise level in your working environment?
   ☐ 1 - Not at all high ☐ 2 ☐ 3 ☐ 4 ☐ 5 – Very High
2. **Frequency of Noise Exposure -** Please rate how often you hear each type of noise at your workstation.

Noise Source	1 – Never	2	3	4	5 – Constantly
**Machine and Equipment**					
Ventilation/air conditioning system	☐	☐	☐	☐	☐
Lighting system	☐	☐	☐	☐	☐
Office machines (computer, copier, keyboard, etc.)	☐	☐	☐	☐	☐
**Human Activity and Interactions**					
Conversations between colleagues	☐	☐	☐	☐	☐
Telephone or Online meeting conversations	☐	☐	☐	☐	☐
Conversations that you cannot fully understand (e.g., distant or muffled speech)	☐	☐	☐	☐	☐
Someone talking to themselves (monologue)	☐	☐	☐	☐	☐
Laughter	☐	☐	☐	☐	☐
People walking/footsteps	☐	☐	☐	☐	☐
Use of furniture (drawers, doors, chairs, desks, etc.)	☐	☐	☐	☐	☐
**Other Background Noise**					
Noise from outside the building (e.g., human activity, equipment, traffic, construction)	☐	☐	☐	☐	☐
Entertainment/music/radio	☐	☐	☐	☐	☐
Kitchen appliances (e.g., microwave, coffee machine)	☐	☐	☐	☐	☐
Others, please specify ______________________	☐	☐	☐	☐	☐

3. **Most Disturbing Noise**
   Among the noises you frequently hear at your workstation, please identify up to three noise sources you find most disturbing. If only one stands out, you may list just one.
   a. ____________ b. ____________ c. ____________

C. Experience of Your Office Sound Environment

1. **Perceived Characteristics of the Sound Environment**
   Please indicate how much you agree or disagree that your current surrounding sound environment has the following characteristics.

Characteristic	1 – Strongly Agree	2	3	4	5 – Strongly Disagree
Comfortable	☐	☐	☐	☐	☐
Pleasant	☐	☐	☐	☐	☐
Promoting Concentration	☐	☐	☐	☐	☐

DRAFT

Characteristic	1 – Strongly Agree	2	3	4	5 – Strongly Disagree
Demotivating	☐	☐	☐	☐	☐
Irritable	☐	☐	☐	☐	☐
Disturbing	☐	☐	☐	☐	☐
Eventful	☐	☐	☐	☐	☐
Vibrant	☐	☐	☐	☐	☐
Full of content	☐	☐	☐	☐	☐
Detached	☐	☐	☐	☐	☐
Uneventful	☐	☐	☐	☐	☐
Empty	☐	☐	☐	☐	☐
Chaotic	☐	☐	☐	☐	☐
Calm	☐	☐	☐	☐	☐
Annoying	☐	☐	☐	☐	☐

2. **Appropriateness of the Sound Environment for Different Tasks**
   How appropriate do you find the current sound environment for the following types of tasks? Please rate your experience accordingly

Task Type	1 – Not at all	2	3	4	5 – Perfectly
Tasks requiring high concentration	☐	☐	☐	☐	☐
Tasks involving discussions or collaboration	☐	☐	☐	☐	☐
Routine or administrative tasks	☐	☐	☐	☐	☐
Creative work requiring inspiration	☐	☐	☐	☐	☐

D. Managing Noise in the Workplace

Please rate your sense of control over noise and how often you use different strategies to cope with it.

1. **Noise Control Measures**
   Are there any noise reduction tools available in your workplace?
   ☐ Yes, and they are effective ☐ Yes, but they are not effective
   ☐ No, they are not available ☐ I am not sure
2. **Sense of Control Over Noise**
   To what extent do you feel you have control over the noise in your workplace?
   ☐ 1 - Not control at all ☐ 2 ☐ 3 ☐ 4 ☐ 5 – Full control
3. **Noise Coping Strategies**
   How do you usually cope with noise in your workplace? *(Select all that apply)*

☐ Listen to music or use a listening device (e.g., earphones, headphones)
☐ Use earplugs or hearing protectors
☐ Take a break and resume work later
☐ Move to a quieter area
☐ Slow down your work pace
☐ Suggest improvement to management
☐ Make an extra effort to stay focused
☐ Work from home
☐ Ask colleagues to lower their voices
☐ Talk to colleagues about the noise
☐ Be quieter in the hope that others follow
☐ Use designated rooms for private calls
☐ Do nothing

DRAFT

E. Your Work Experience and Office Noise

Please rate your level of satisfaction and experiences regarding communication, workload and emotions in your workplace.

1. Communication and Office Sound

How satisfied are you with workplace communication under the current noise conditions?

Statement	1 - Very dissatisfied	2	3	4	5	6	7 - Very satisfied
Talking to colleagues about work	☐	☐	☐	☐	☐	☐	☐
Having casual conversations with colleagues	☐	☐	☐	☐	☐	☐	☐
Making phone calls to colleagues	☐	☐	☐	☐	☐	☐	☐

2. Workload, Emotion and Office Sound

How often do the following situations occur due to noise in your workplace?

Statement	1 - Never	2	3	4	5 – Always
I still have enough time for my work tasks, despite the noise in my office. (R)	☐	☐	☐	☐	☐
I get behind with my work due to noise distractions in my office.	☐	☐	☐	☐	☐
I work at a high pace throughout the day because of the noise in my office.	☐	☐	☐	☐	☐
Noise in my office makes it necessary to keep working at a high pace.	☐	☐	☐	☐	☐
Noise in my office causes emotional discomfort or distress.	☐	☐	☐	☐	☐
Noise in my office makes it difficult for me to engage emotionally with others.	☐	☐	☐	☐	☐

Note: This draft questionnaire was developed based on findings from the previous systematic review [1] and insights gathered during the focus group discussion. All citations included in the draft questionnaire are clearly explained in the main manuscript.

DRAFT

Reference:

[1] Rachman Z, Aletta F, Kang J. Exploring Soundscape Assessment Methods in Office Environments: A Systematic Review. *Buildings* 2024; 14: 3408.
